# Autophagy diminishes the early interferon-β response to influenza A virus resulting in differential expression of interferon-stimulated genes

**DOI:** 10.1038/s41419-018-0546-5

**Published:** 2018-05-10

**Authors:** Brieuc P. Perot, Jeremy Boussier, Nader Yatim, Jeremy S. Rossman, Molly A. Ingersoll, Matthew L. Albert

**Affiliations:** 1 Unit of Dendritic Cell Immunobiology, Department of Immunology, Institut Pasteur, Paris, France; 2 Inserm 1223, Paris, France; 3 Ecole Doctorale Physiologie, Physiopathologie et Thérapeutique, Université Pierre et Marie Curie (Université Paris 6), Paris, France; 4 International Group for Data Analysis, Institut Pasteur, Paris, France; 5 Ecole Doctorale Frontières du Vivant, Université Paris Diderot, Paris, France; 6 School of Biosciences, University of Kent, Canterbury, UK; 7 Department of Cancer Immunology, Genentech Inc., South San Francisco, CA USA

## Abstract

Influenza A virus (IAV) infection perturbs metabolic pathways such as autophagy, a stress-induced catabolic pathway that crosstalks with cellular inflammatory responses. However, the impact of autophagy perturbation on IAV gene expression or host cell responses remains disputed. Discrepant results may be a reflection of in vivo studies using cell-specific autophagy-related (*Atg*) gene-deficient mouse strains, which do not delineate modification of developmental programmes from more proximal effects on inflammatory response. In vitro experiments can be confounded by gene expression divergence in wild-type cultivated cell lines, as compared to those experiencing long-term absence of autophagy. With the goal to investigate cellular processes within cells that are competent or incompetent for autophagy, we generated a novel experimental cell line in which autophagy can be restored by ATG5 protein stabilization in an otherwise *Atg5*-deficient background. We confirmed that IAV induced autophagosome formation and p62 accumulation in infected cells and demonstrated that perturbation of autophagy did not impact viral infection or replication in ATG5-stablized cells. Notably, the induction of interferon-stimulated genes (ISGs) by IAV was diminished when cells were autophagy competent. We further demonstrated that, in the absence of ATG5, IAV-induced interferon-β (IFN-β) expression was increased as compared to levels in autophagy-competent lines, a mechanism that was independent of IAV non-structural protein 1. In sum, we report that induction of autophagy by IAV infection reduces ISG expression in infected cells by limiting IFN-β expression, which may benefit viral replication and spread.

## Introduction

Macroautophagy (hereafter referred to as autophagy) is a catabolic pathway conserved among eukaryotes by which cytoplasmic elements are isolated within double-membrane autophagosomes that mature by fusing with the endo-lysosomal compartment^1^. The elongation of autophagosome membranes requires two ubiquitin-like conjugation systems^1^. First, autophagy-related 5 (ATG5) is conjugated to ATG12, which is required for formation of the second complex composed of phosphatidylethanolamine (PE) conjugated to microtubule-associated protein 1 light chain-3 (LC3). Free cytosolic LC3 is referred to as LC3-I, whereas the PE-conjugated form is termed LC3-II. Autophagy occurs in all nucleated cells, playing a key role in maintaining homeostasis^2^. In stress conditions, such as viral infection, autophagic activity may increase^3–5^.

Autophagy can increase or decrease viral fitness depending on the virus or model system studied^6^. One direct antiviral action mediated by autophagy is the degradation of viral components^7^. Autophagy can also alter antiviral cell pathways, including programmed cell death, sensing of virus-associated molecular patterns and cytokine secretion^4, 8^. For example, autophagy supports hepatitis C virus (HCV) replication and negatively regulates interferon-β (IFN-β) induction during HCV infection^9–12^.

Influenza A virus (IAV), a member of the *Orthomyxoviridae* family, causes yearly epidemic infections and sporadic pandemics. IAV-related symptoms are mainly the result of excessive inflammation including high levels of pro-inflammatory cytokines^13^. IAV virus-associated molecular patterns are sensed by Toll-like receptors, nucleotide oligomerization domain-like receptors and retinoic acid-induced gene I (RIG-I)-like receptors, which induce type I IFN and pro-inflammatory cytokine secretion^14^. IAV has evolved mechanisms to antagonize innate immune responses in infected cells, mainly via non-structural protein 1 (NS1), which inhibits RIG-I signalling^14^. While IAV stimulates autophagy, its matrix protein 2 (M2) has been proposed to block the maturation of autophagosomes, although this finding remains disputed^15–18^.

We investigated the impact of IAV-mediated autophagy perturbation on the host cell response to infection. We designed our study to circumvent limitations of techniques commonly used to study autophagy. Notably, chemical treatments used to manipulate autophagy impact other biological processes. For example, rapamycin, used to inhibit autophagy, inhibits the kinase activity of the mammalian target of rapamycin, impacting transcription, translation and mitochondrial metabolism^19^. Transfection of small interfering RNAs (siRNAs) to suppress autophagy genes can activate innate signalling pathways in a structure- or sequence-dependent manner^20^. Knockout (KO) or siRNA knockdown cell lines are subject to genetic drift, with compensatory mutations resulting in unanticipated off-target effects when compared to wild-type (WT) cell lines^21–23^. Finally, the ATG5 tet-off cell system is prone to bias due to the requirement of long-term exposure to doxycycline to repress autophagy^24^. Notably, doxycycline and related antibiotics can alter mitochondrial function, inflammation, proliferation, metabolism and, in some instances, induce cell death^25–33^.

We generated a new experimental model in which the capacity to undergo autophagy can be controlled through drug-induced stabilization of critical components of the autophagy pathway that are otherwise targeted for degradation. Importantly, this model does not induce autophagy but instead restores the capacity of a cell to undergo autophagy. We observed that autophagy was dispensable for IAV replication, but cells lacking a functional autophagy pathway had an enhanced type I IFN-induced inflammatory response at early time points post-infection. Together, our findings clarify the interplay of IAV infection, autophagy and host response. Moreover, the experimental model presented herein will establish a new path towards validating the role of autophagy during inflammatory processes.

## Results

### A novel model to initiate autophagy through the induced stabilization of ATG5

**Fig. 1:**
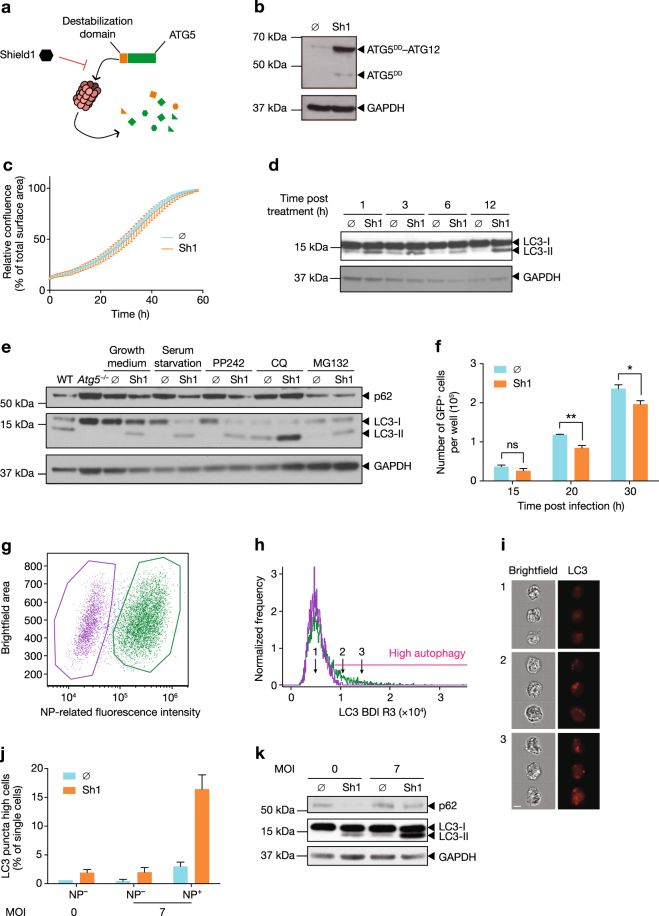
Stabilization of ATG5 in *Atg5*^*–/–*^ cells enables experimental control of autophagy. **a** Schematic representation of Shield1 (Sh1) stabilization of ATG5 illustrates the rescue of destabilization domain (DD)-fused ATG5 (ATG5^DD^). **b** ATG5^DD^-expressing *Atg5*^*–/–*^ cells were treated with ethanol vehicle (∅) or Sh1 for 20 h, followed by immunoblot analysis with anti-ATG5 antibody. **c** ATG5^DD^-expressing *Atg5*^*–/–*^ cells were treated with Sh1 and images were obtained every hour for 60 h to assess cell growth. Points depict mean confluence at interval time and error bars depict standard deviation. **d** ATG5^DD^-expressing cells were treated for the indicated times with Sh1 or vehicle (∅). Protein extracts were subjected to immunoblot analysis using anti-LC3 and anti-GAPDH antibodies. **e** In the presence or absence of Sh1, cells were exposed to serum deprivation, an inhibitor of the mammalian target of rapamycin (PP242), a proton pump inhibitor (chloroquine, CQ) or a proteasome inhibitor (MG132). Wild-type (WT) and *Atg5*^*–/–*^ MEFs (*Atg5*^*–/–*^*)* were used as positive and negative controls, respectively. After 4 h of culture, protein extracts were subjected to immunoblot analysis using anti-p62, anti-LC3 and anti-GAPDH antibodies. **f** ATG5^DD^ cells, pretreated or not with Shield1 (Sh1), were infected with GFP-expressing chikungunya virus at an MOI of 0.1. The number of green cells were monitored through live imaging. Graph shows mean and standard deviation of three biological replicates, and data are representative of three experiments. ns, not significant; *q < 0.01, **q < 0.01 (two-tailed unpaired *t*-test followed by Holm’s multiple testing correction). **g**–**k** ATG5^DD^ expression was stabilized by Sh1 treatment and cells were infected with influenza A virus (IAV). After 20 h, cells were fixed, permeabilized and stained using anti-NP antibody to quantify IAV infection and anti-LC3 antibody to visualize autophagy puncta. Imaging flow cytometry permitted gating based on NP expression (representative dot plot, **g**) and quantification of autophagic vesicles using the bright detail intensity R3 (BDI R3, histogram, **h**). **i** Three representative images of single cells with three different BDI R3, corresponding to the indicated numbered arrows in **h**, are shown. The topography of LC3 within cells is shown in red. Scale bar, 10 μm. **j** Graphs plot mean percentage of cells with high autophagic vesicle content and standard deviation; analysis is based on images captured from >10,000 cells per experiment (*n* = 2 experiments). **k** Cells were analysed by immunoblot for p62, LC3 and GAPDH expression

Many experimental systems used to study autophagy result in off-target effects due to the disruption of bystander pathways. To avoid potential confounding artefacts, we generated novel expression systems and cell lines in which autophagy can be controlled through the induced stabilization of ATG5. We generated clonal populations of *Atg5*^–/–^ mouse embryonic fibroblasts (MEFs) that stably expressed the ATG5 protein fused to a destabilization domain (ATG5^DD^), which is known to be rapidly degraded by the proteasome (Fig. 1a)^34^. The small, biologically inert, cell-permeable molecule, Shield1, interacts with the destabilization domain, preventing its degradation by the proteasome^34, 35^. Addition of Shield1 to *Atg5*^–/–^ MEFs expressing ATG5^DD^ rescued the degradation of the ATG5^DD^ fusion protein (Fig. 1b). Notably, accumulated ATG5^DD^ protein was primarily found within ATG5–ATG12 complexes, supporting that the fusion protein retained this function (Fig. 1b). Intra-incubator microscopy was used to measure cell confluence over time, with or without the addition of Shield1, revealing that ATG5^DD^ accumulation, in nutrient-rich conditions, did not impact the kinetics of cell growth (Fig. 1c).

We next tested whether ATG5^DD^ stabilization restored the ability of the cell to undergo autophagy, as determined by the conversion of LC3-I to LC3-II, a measure of autophagosome and autophagolysosome accumulation^36^. Shield1 rescued basal autophagy as early as 1 h post addition (Fig. 1d). As expected, untreated ATG5^DD^ cells were phenotypically similar to the parental *Atg5*^*–/–*^ cell line. Both lines exhibited low levels of LC3-II conversion; however, following Shield1 treatment, modest levels of LC3-II could be detected in the ATG5^DD^ cell line, similar to the levels of autophagy in WT cells (Fig. 1e). Furthermore, we observed that inducing autophagy by serum starvation or PP242 treatment or inhibiting autophagolysosome function using chloroquine led to increased LC3-II/LC3-I ratios within Shield1-treated cells (Fig. 1e). We also measured p62 expression, an adaptor protein that is degraded in the course of autophagy and whose accumulation in the presence of high levels of LC3-II is indicative of abortive autophagy^36^. We demonstrated that p62 levels were reduced following Shield1 treatment, with further reduction observed in cells exposed to serum starvation or PP242 (Fig. 1e). As expected, p62 was not degraded in autophagy-competent cells that were treated with chloroquine, which blocks autophagosome fusion with lysosomes^36^. As the DD is expected to lead to proteasomal degradation of ATG5^DD^, we tested whether proteasome inhibition by MG132 would result in Shield1-independent ATG5^DD^ accumulation and rescue of autophagy competence. Indeed, MG132 treatment resulted in p62 degradation in both control and Shield1-treated cells (Fig. 1e). Together, these data established that ATG5^DD^ was degraded by the proteasome and that Shield1 treatment restored the capacity to undergo autophagy in ATG5^DD^-expressing *Atg5*^*–/–*^ cells. We validated that our cell-based system rescued autophagy by testing whether chikungunya virus (CHIKV) propagation was inhibited upon Shield1 treatment (Fig. 1f)^37^.

We next assessed autophagy in the context of IAV infection, exposing ATG5^DD^ cells to IAV A/PR/8/1934 (H1N1, PR8 strain) for 20 h in the presence or absence of Shield1. Using imaging flow cytometry, we delineated infected and uninfected cells (Fig. 1g) and measured autophagic cells within each population (Fig. 1h). IAV-induced LC3 puncta accumulated in IAV-infected cells (green) but not in uninfected cells (purple) (Fig. 1i, j). Overall, LC3-II levels increased when Shield1 was present during infection (Fig. 1k). Notably, p62 was not degraded following Shield1 treatment during infection, consistent with previous reports that IAV inhibits autophagosome maturation^17^.

### Autophagy does not impact viral infection or replication

**Fig. 2:**
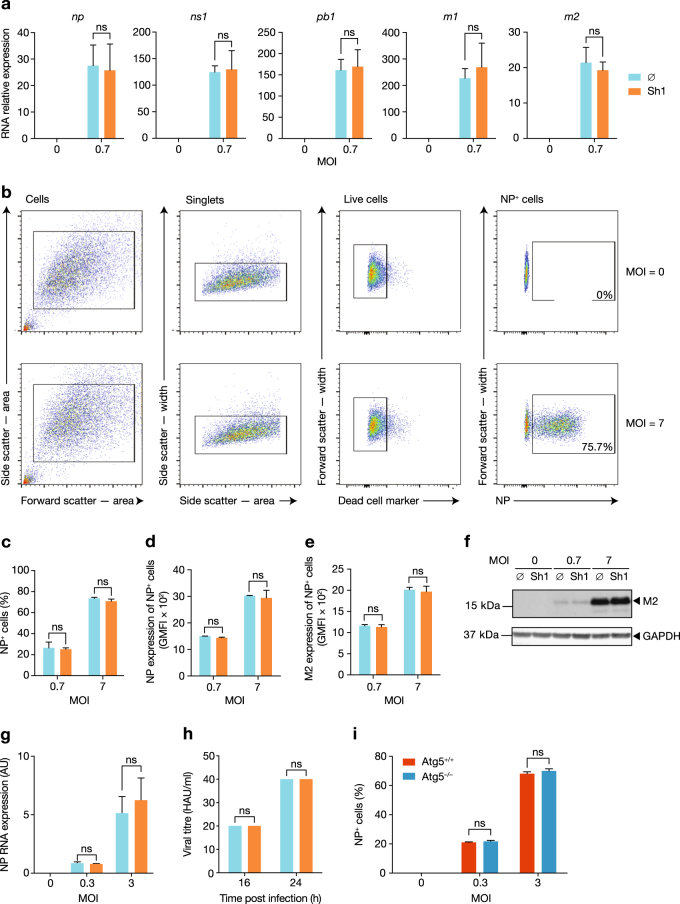
ATG5 stabilization does not impact IAV replication. **a** Following 5 h infection, with or without Shield1 (Sh1) pretreatment, IAV RNA expression levels were determined using RT and qPCR primer/probe sets specific for the NP, NS1, PB1, M1 and M2 genes. **b**–**d** ATG5^DD^ cells, with or without Sh1 treatment, were infected with IAV for 16 h at the indicated MOI. The gating strategy after nucleoprotein (NP) immunostaining and flow cytometry is shown for two samples: uninfected, and infected at an MOI of 7 (**b**). The percentage of NP-expressing cells (**c**) and the geometric mean fluorescent intensity (GMFI) of NP per cell (**d**) were determined by flow cytometry. **e**, **f** IAV M2 expression was determined using flow cytometry (**e**) and immunoblotting (**f**) following 16 or 20 h infection at the indicated MOI, respectively. **g** ATG5^DD^ cells, pretreated or not with Shield1 (Sh1), were infected for 16 h at the indicated MOI. NP RNA expression in supernatants was analysed by RT–qPCR. **h** ATG5^DD^ cells, pretreated or not with Shield1 (Sh1), were infected at MOI 3 for the indicated times; haemagglutination assays were used to quantify of the number of haemagglutinin units (HAUs) per millilitre of supernatant. **i** *Atg5*^+/+^ or *Atg5*^–/–^ were infected with IAV at the indicated MOI for 16 h. Percentages of NP-expressing cells was determined by flow cytometry. Graphs show mean and standard deviation of biological triplicates, and data are representative of two experiments. ns, not significant (one-tailed unpaired *t*-test followed by Holm’s multiple testing correction)

As autophagy impacts viral replication in several infectious models^7, 38^, we used our inducible cell lines to investigate the influence of autophagy on IAV replication. We found that the IAV RNA content of infected samples at 5 h post-infection was not different between control and Shield1-treated cells (Fig. 2a). The expression of nucleoprotein (NP) in infected cells was measured by flow cytometry (Fig. 2b). The percentage of NP-expressing cells was similar between control and Shield1-treated cells 16 h post-infection, at low and higher multiplicities of infection (MOIs) (Fig. 2c). Moreover, the intensity of NP expression within IAV-infected cells was unchanged (Fig. 2d). M2 protein was also expressed at similar levels (Fig. 2e, f). Finally, haemagglutinin protein and NP RNA levels in supernatants were similar in autophagy-competent and -incompetent cells (Fig. 2g, h). Of note, low levels of LC3-II were observed in the absence of Shield1 when cells were infected by IAV (Fig. 1j). This is likely a result of lower levels of proteasome activity, which in turn permitted modest Atg5 expression. To confirm that low levels of autophagy were not required for IAV replication, we infected *Atg5*^–/–^ MEFs, showing that autophagy is indeed dispensable for infection (Fig. 2i). Taken together, these data demonstrate that restoration of autophagy did not impact IAV infection or replication.

### Autophagy limits interferon-stimulated gene (ISG) induction independently of the key IAV anti-type I IFN protein NS1

**Fig. 3:**
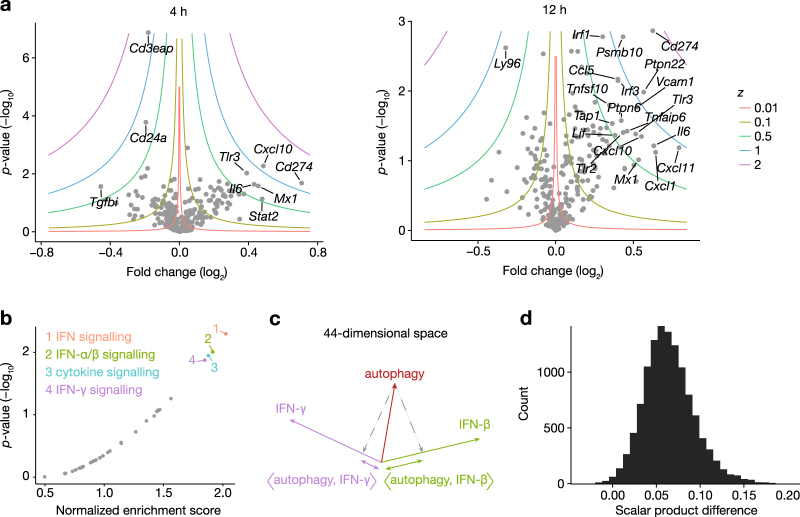
IAV-induced expression of type I IFN-stimulated genes is reduced when ATG5^DD^ is stabilized. **a** ATG5^DD^ cells, pretreated for 16 h with Shield1 (Sh1), were infected with IAV PR8 at MOI 3 for 4 or 12 h followed by RNA extraction. mRNA levels of 561 genes (see Materials and methods) were quantified using Nanostring nCounter technology. Volcano plots show the *p*-value determined by two-tailed paired *t*-tests and fold change of gene expression in control vs. Sh1-treated cells. Iso *z*-value curves are depicted. Data were generated from three independent experiments. **b** Gene set enrichment analysis was performed after ranking genes according to their differential expression in control vs. Sh1-treated samples (see Materials and methods for computation of *t*-statistic). Shown are normalized enrichment score and *p*-values, computed by the GSEA method^40^. Each point represents a gene set (the 40 most enriched gene sets are shown), and sets with an enrichment false-discovery rate <0.2 are coloured and labelled. **c** The 44 genes most significantly impacted by Sh1 treatment (paired *t*-test *p*-value <0.05) were selected and weighted by their *t*-statistic, which gave rise to an “autophagy” vector lying in a 44-dimensional space (red). Data from whole-blood stimulation were used to create an IFN-β vector (control vs. IFN-β treatment *t*-statistic, green) and an IFN-γ vector (control vs. IFN-β treatment t-statistic, purple), after which all vectors were normalized to length 1. **d** Bootstrapping over the 25 donors of the whole-blood study was performed and the difference 〈autophagy, IFN-β〉−〈autophagy, IFN-γ〉 between the two scalar products was computed for each iteration. Plotted is the distribution of the differences, with a 95% confidence interval of (0.014, 0.12)

We next wanted to determine whether autophagy impacts the host response to IAV. To test whether immune pathways were impacted by autophagy, we used the NanoString nCounter technology, a method that allows the quantitative measurement of single mRNA molecules, without the need for RT or amplification. We measured RNA from a 561 gene set of immunology-related genes, including cytokine and Toll-like receptor-related pathways (Supplementary Figure S1). ATG5^DD^ cells, pretreated or not with Shield1 for 16 h, were infected with IAV for 4 or 12 h. Raw counts were normalized to the geometric mean value of five internal control genes (*Ppia*, *Gapdh*, *Rpl19*, *Oaz1* and *Polr2a*), selected based on the application of the geNorm method^39^ (Supplementary Figure S2 and Materials and methods). From the results of three independent experiments, we found that the inability to undergo autophagy during infection led to higher expression of many pro-inflammatory genes as compared to autophagy-competent cells, including established ISGs, such as *Psmb10*, *Cd274*, *Cxcl10*, *Irf1* and *Tap1* (Fig. 3a and Supplementary Table S1). Of note, two members of the class I major histocompatibility complex (MHC I) pathway, *Psmb10* and *Tap1*, were decreased in autophagy-competent cells (Fig. 3a). To identify biological processes and signalling pathways, rather than single genes, impacted by autophagy capacity in a robust fashion, we performed a gene set enrichment analysis (GSEA)^40^. Among the 129 gene sets tested (corresponding to sets from databases that included at least five genes measured in this experiment and with expression levels consistently above the lower limit of quantification), we identified four pathways that were significantly enriched in genes differentially expressed by autophagy-competent (Shield1-treated) vs. autophagy-deficient (control vehicle-treated) infected cells (Fig. 3b), which were ranked by decreasing order of enrichment: IFN signalling; IFN-α/β signalling; cytokine signalling; and IFN-γ signalling. Owing to shared gene expression among IFN-γ and IFN-α/β signalling pathways, and given that GSEA relies on unweighted gene set lists, this method is not suitable for distinguishing between type I and type II IFN responses. We, therefore, implemented a more quantitative approach, using recently published data comparing the gene signature of IFN-β- and IFN-γ-stimulated whole blood, analysed by the same nCounter technology^41^. The genes most significantly impacted by autophagy and present in the gene sets of both our study and the whole blood approach (44 genes, *t*-test *p*-value < 0.05, Supplementary Table S1) were weighted by their *t*-statistic, which gave rise to an “autophagy” vector lying in a 44-gene feature dimensional space. We then used data from control, IFN-β or IFN-γ whole-blood stimulation of 25 healthy donors to create two new vectors by weighting each gene by its *t*-statistic (paired *t*-test, control vs. IFN-β or control vs. IFN-γ). All vectors were normalized to length 1, and the “autophagy” vector was projected onto the “IFN-β” and “IFN-γ” vectors (Fig. 3c). The scalar product 〈autophagy, IFN-β〉 was found to be greater than the 〈autophagy, IFN-γ〉 scalar product. Bootstrapping over the 25 donors from the whole-blood study confirmed the robustness of this result, as the difference between the two scalar products showed a consistent positive value (95% confidence interval = (0.014, 0.12)), suggesting that the autophagy signature was more characteristic of stimulation by IFN-β than by IFN-γ (Fig. 3d). Moreover, *Ifnb1* was the only IFN gene consistently detected in our cellular model after infection (Supplementary Figure S3). These results indicate that autophagy modulates IFN-β-stimulated genes following IAV infection.

**Fig. 4:**
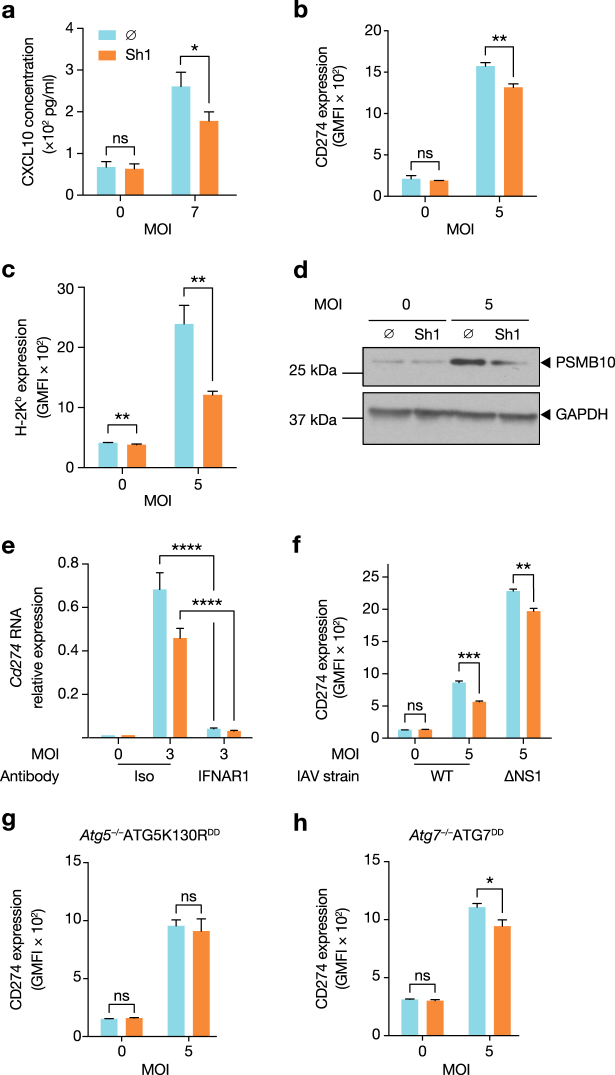
ISG expression levels are suppressed by autophagy machinery during IAV infection. **a** ATG5^DD^ cells, pretreated or not with Shield1 (Sh1) for 16 h, were infected with IAV at the indicated MOIs for 5 h. CXCL10 concentration in the supernatants was measured by ELISA. **b**, **c** ATG5^DD^ cells, pretreated or not with Sh1 for 20 h, were infected with IAV at the indicated MOIs for 16 h. Surface expression of CD274 (**b**) and H-2K^b^ (**c**) was measured by flow cytometry. **d** ATG5^DD^ cells, pretreated or not with Sh1 for 20 h, were infected with IAV PR8 at the indicated MOIs for 20 h before analysing PSMB10 expression by immunoblot. **e** ATG5^DD^ cells, pretreated or not with Sh1 for 16 h and with anti-IFNAR1 antibody for 1 h, were infected for 5 h at the indicated MOIs and *Cd274* expression was assayed by RT–qPCR (calculated as 2^(CtHprt1−CtCd274)^). **f** ATG5^DD^ cells, pretreated or not with Sh1 for 20 h, were infected with PR8 or ΔNS1 PR8 at the indicated MOIs for 16 h before measuring CD274 surface expression by flow cytometry. **g**, **h** ATG5K130R^DD^ (**g**) or ATG7^DD^ (**h**) cells were treated as in **b** and **c** before monitoring of surface CD274 expression by flow cytometry. **a**–**c**, **e**–**h** Graphs show mean and standard deviation of three biological replicates, and data are representative of three experiments. ns, not significant; *q < 0.05, **q < 0.01, ***q < 0.001, ****q < 0.0001 (one-tailed unpaired *t*-test followed by Holm’s multiple testing correction)

We next tested whether IAV-induced autophagy impacted the inflammatory response at the protein level. During IAV infection, C-X-C chemokine motif ligand 10 (CXCL10) secretion was decreased by ATG5^DD^ stabilization, with differential expression between Shield1-treated and -untreated cells detected as early as 5 h (Fig. 4a). Furthermore, surface expression of CD274 and class I major histocompatibility protein H-2K^b^ were reduced by ATG5^DD^ stabilization 16 h post-infection (Fig. 4b, c). Of note, surface expression of H-2K^b^ was also significantly, although to a lesser extent, impacted by autophagy in uninfected cells. Additionally, we measured PSMB10 expression, a subunit of the immunoproteasome, which was also reduced in autophagy-competent cells (Fig. 4d). All three of these proteins are induced by type I IFN^42–45^ and support the conclusion that IAV-induced autophagy negatively regulates IFN-β–induced inflammatory responses. We confirmed that *Cd274* was indeed an ISG by blocking IFN-α/β receptor (IFNAR) signalling through the use of a neutralizing anti-IFNAR1 antibody (Fig. 4e). *Cd274* was the most differentially expressed ISG at the RNA level. Therefore, we used this molecule, as well as H-2K^b^ expression, as functional readouts of the impact of autophagy for the remainder of our study. As autophagy limits vesicular stomatitis virus (VSV)-induced inflammation through dampening of cellular reactive oxygen species (ROS) content^46^, we tested whether restoring autophagy capacity led to changes in cellular ROS content. Measurement of both total and mitochondrial ROS revealed that Shield1 treatment did not impact ROS content, arguing that differences in ROS are not responsible for the hyperinflammatory phenotype of autophagy-incompetent cells (Supplementary Figure S4). We next investigated whether NS1, a key negative regulator of the type I IFN response^47^, played a role in the suppression of ISGs following infection. We infected cells with wild type and ΔNS1 IAV PR8 in the presence or absence of Shield1. Stabilization of ATG5^DD^ decreased CD274 expression following infection with either viral strain (Fig. 4f).

ATG5 mediates several non-autophagy-related phenotypes^48^. Thus we generated an *Atg5*^–/–^ cell line that stably expressed the mutant ATG5 molecule, ATG5K130R^DD^. ATG5 lysine K130 is an amino acid required for binding to ATG12, thus ATG5K130R cannot form the ATG5–ATG12 complex. We confirmed that ATG5K130R^DD^ accumulated upon Shield1 treatment (Supplementary Figure S5). However, as expected, we did not detect a band corresponding to the ATG5–ATG12 complex in Shield1-treated cells. LC3-II was also undetectable (Supplementary Figure S5). We infected ATG5K130R^DD^ cells in the presence or absence of Shield1 and observed that lysine 130 of ATG5 was required for suppression of CD274 expression (Fig. 4g). We established *Atg7*^–/–^ cell lines, which stably expressed ATG7^DD^, thus permitting controlled regulation of a distinct step in autophagy. The ATG7 protein is required for ATG5 conjugation to ATG12 during autophagosomal membrane elongation^49^. Confirming that ATG7 was stabilized, Shield1 treatment permitted ATG5–ATG12 complex formation, rendering the cell line competent for autophagy as measured by the LC3-II/LC3-I ratio (Supplementary Figure S5). Validating our findings in the ATG5^DD^ cell line, we demonstrated that ATG7^DD^ stabilization also resulted in reduced CD274 expression in infected cells (Fig. 4h). Together, we conclude that ATG5–ATG12 complex formation is a key step in limiting ISG induction in response to IAV infection.

### Early post-infection, virus-induced autophagy decreases IFN-β expression leading to diminished ISG expression

**Fig. 5:**
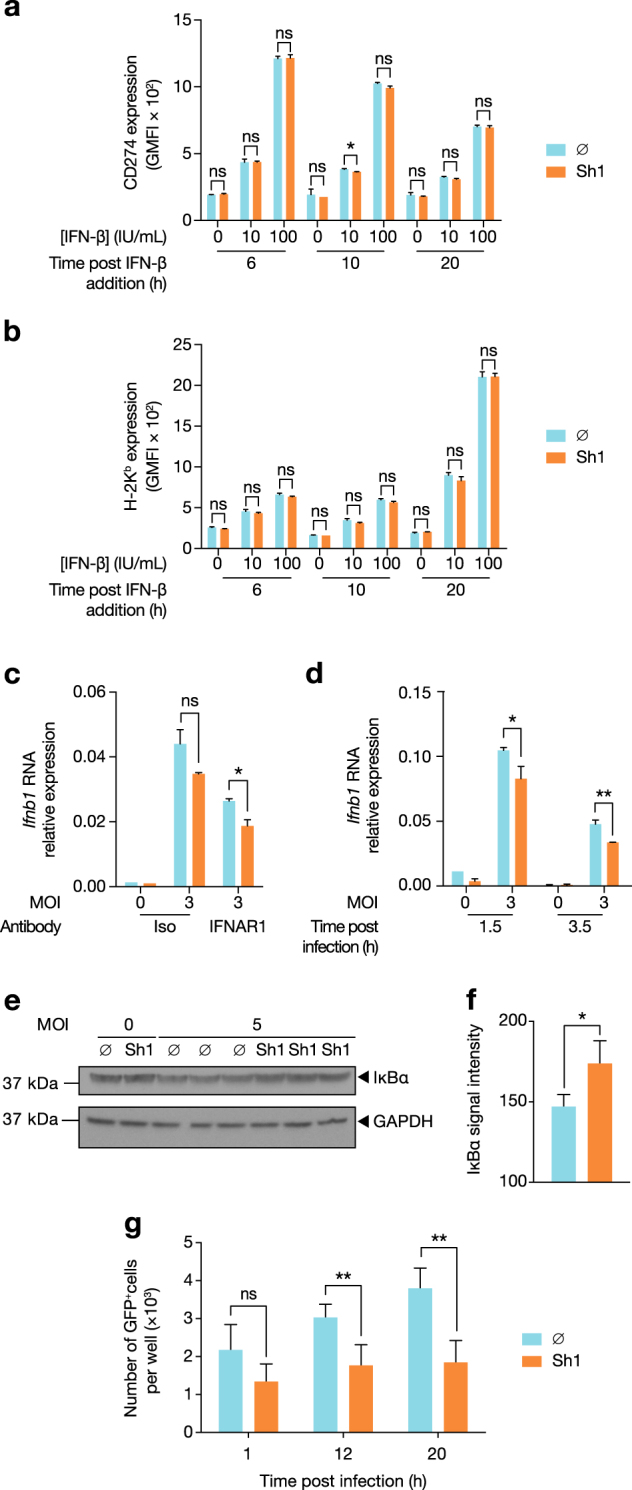
ATG5^DD^ stabilization inhibits ISG expression via cell-intrinsic modulation of IFN-β expression and not through desensitization to IFNAR signalling. **a**, **b** ATG5^DD^ cells, pretreated or not with Shield1 (Sh1), were treated with recombinant mouse IFN-β at the indicated concentration for 6, 10 or 20 h, followed by surface expression analysis of CD274 (**a**) and H-2K^b^ (**b**) by flow cytometry. **c** ATG5 ^DD^ cells were pretreated with anti-IFNAR1 antibodies followed by IAV infection. *Ifnb1* expression was assayed by RT–qPCR. **d** In independent experiments, *Ifnb1* induction was measured at 1.5 and 3.5 h post-infection in autophagy-competent (Sh1) vs. autophagy-null cells (∅). **e**, **f** ATG5^DD^ cells, pretreated or not with Shield1 (Sh1), were infected with IAV for 1 h before monitoring IκBα degradation by immunoblot (**e**) and quantification with ImageJ (**f**). **g** ATG5^DD^ cells transfected with NF-κB transcription activity GFP reporter for 20 h were pretreated or not with Shield1 (Sh1) and infected with IAV. Incucyte intra-incubator microscope allowed the monitoring of GFP-positive cells. **a**–**d**, **f**, **g** Graphs show mean and standard deviation of three (**a**–**d**, **f**) or four (**g**) biological replicates, and data are representative of three experiments. ns, not significant; *q < 0.05, **q < 0.01 (one-tailed unpaired *t*-test followed by Holm’s multiple testing correction)

**Fig. 6:**
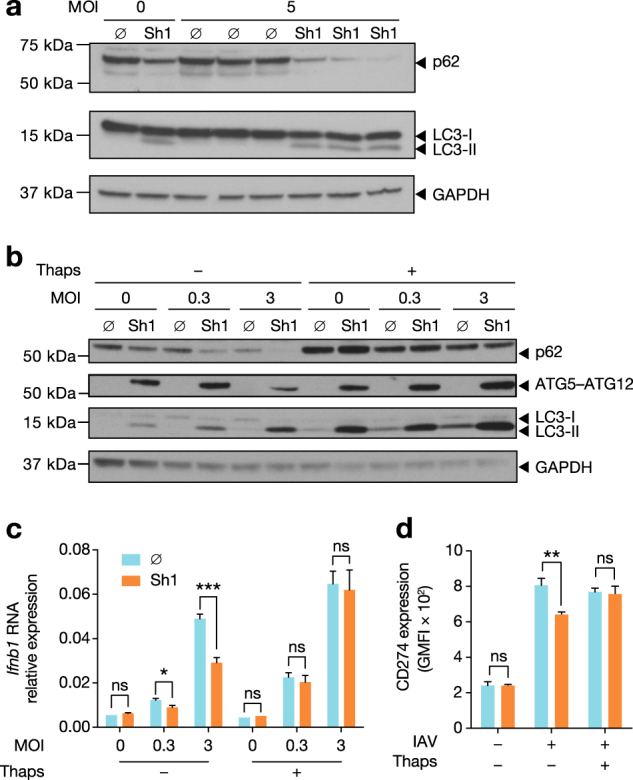
Autophagy must go to completion to inhibit IFN-β expression. **a** ATG5^DD^ cells were pretreated or not with Sh1 for 16 h and infected with IAV at the indicated MOIs. One hour post-infection, p62, LC3-I, LC3-II and GAPDH levels were measured by immunoblot. Three biological replicates were run for infected control or Sh1-treated samples. **b** ATG5^DD^ cells were pretreated or not with Sh1 for 16 h and infected with IAV at the indicated MOIs, with or without thapsigargin (Thaps) for 5 h. p62, LC3-I, LC3-II, ATG5–ATG12 and GAPDH levels were measured by immunoblot. **c** ATG5^DD^ cells were pretreated or not with Sh1 and infected with IAV at the indicated MOIs, with or without thapsigargin. Three hours post-infection, *Ifnb1* expression was assayed by RT–qPCR. **d** ATG5^DD^ cells were pretreated or not with Sh1 and infected with IAV. Sixteen hours post-infection, surface CD274 expression was monitored by flow cytometry. **c**, **d** Graphs show mean and standard deviation of three biological replicates, and data are representative of three experiments. ns, not significant; *q < 0.05, **q < 0.01, ***q < 0.001 (one-tailed unpaired ∅ followed by Holm’s multiple testing correction)

Based on the differential expression of ISGs, we considered two possible hypotheses: that autophagy negatively impacted IFNAR signalling or, alternatively, that IAV-infected, autophagy-competent cells produced less IFN-β. To address the first possibility, we directly tested whether autophagic flux impacted IFNAR signalling. Following treatment with Shield1 or vehicle for 16 h, ATG5^DD^ cells were exposed to increasing concentrations of recombinant IFN-β. CD274 and H-2K^b^ expression was equally increased following IFN-β treatment, suggesting that ATG5^DD^ stabilization did not alter IFNAR signalling (Fig. 5a, b). To test our alternate hypothesis, we treated cells with IFNAR blocking antibody and observed by reverse transcription–quantitative polymerase chain reaction (RT–qPCR) that ATG5^DD^ stabilization inhibited *Ifnb1* expression independently of IFNAR signalling (Fig. 5c). Importantly, we previously determined that RT–qPCR was sensitive enough to detect fold changes as small as 1.4 with 80% power using triplicate samples, thus permitting confirmation of the results we observed in the nCounter analysis (Supplementary Figure S6). ATG5^DD^ stabilization resulted in reduced IFN-β expression as early as 1.5 h post-infection (Fig. 5d). Early IFN-β expression in the context of virus infection relies on nuclear factor-kappaB (NF-κB) activation^50^. To determine the upstream signalling events impacted by autophagy, we measured NF-κB activation through IκBα degradation and NF-κB reporter assay, observing that autophagy limited IAV-induced NF-κB activation (Fig. 5e–g). We then tested whether autophagy was induced by IAV at this early time point. LC3-II/LC3-I ratio was increased at 1 h post-infection correlating with decreased levels of p62 when Shield1 was present. These findings argue that autophagy was rapidly induced and that IAV-mediated inhibition of autophagy maturation occurred later in the viral life cycle (Fig. 6a, see also Fig. 1j). Interestingly, the ATG5–ATG12 complex limits VSV-induced type I IFN^51^. We assessed whether the ATG5–ATG12 complex was sufficient to modulate type I IFN or, alternatively, whether the effect required autophagosome maturation. We treated cells with thapsigargin, which blocks autophagosome maturation without preventing ATG5–ATG12 conjugation^52^. As expected, thapsigargin treatment potentiated LC3-II accumulation and prevented p62 degradation in cells where ATG5^DD^ was stabilized (Fig. 6b) but did not inhibit ATG5^DD^–ATG12 complex formation (Fig. 6b). Notably, treatment with thapsigargin abrogated the negative impact of autophagy on the expression of *Ifnb1* and induced expression of CD274 (Fig. 6c, d). Thus we concluded that induction of autophagy by IAV at early time points in the viral life cycle inhibits IFN-β induction. This inhibitory effect was independent of NS1 and required autophagosome maturation.

## Discussion

Our study describes the generation of a cellular model in which the capacity of a cell to undergo autophagy can be dynamically restored within 1 h after addition of a small, immunologically inert molecule. Currently available models, on the contrary, do not enable rapid manipulation of the autophagy pathway and are confounded by off-target effects^24–33, 53–55^. The development of a rapid autophagy restoration system permitted us to dissect the impact of autophagy perturbation on inflammatory responses to viral infection. One important caveat, however, is that, under selected conditions, we observed low levels of ATG5^DD^ and autophagic activity in cells in which we did not rescue the destabilized ATG5^DD^. However, cells in which ATG5^DD^ was stabilized through Shield1 had autophagy levels that were comparable to that of WT MEFs, and untreated cells exhibited a marked decrease in autophagic activity.

Using this model system, we demonstrated that the absence of ATG5 did not impact IAV infectivity or replication, yet the induction of ISGs, such as CD274 and MHC I, were diminished as a result of IAV-induced autophagy. With respect to regulation of MHC I, we suggest that the negative impact of autophagy (secondary to ATG5^DD^ stabilization) is mediated by decreased expression of two MHC I presentation pathway genes, *Psmb10* and *Tap1* (see also Table S1). These findings are in agreement with a report that autophagy-deficient dendritic cells express higher levels of MHC I on their surface at steady state and more efficiently prime anti-IAV-specific CD8^+^ T-cell responses^56^. Of note, even though autophagy limited the induction of ISGs, we did not detect any impact on IAV replication at later time points. This may be a reflection of a key anti-IAV ISG, MX1, being inactive in our cells^57, 58^. Alternatively, we may not observe the impact of ISGs due to our experimental approach focussing on the first cycle of replication, a direct result MEFs lacking the protease activity required cleave haemagglutinin and generate replication competent viral progeny^59, 60^.

The decreased expression of key proteins involved in immune regulation correlated with decreased of *Ifnb1* expression and lower NF-κB activation. Interestingly, the best-described mechanism by which IAV inhibits pattern recognition receptor signalling and subsequent IFN-β and ISG induction is through the action of NS1^61–63^. Using IAV lacking expression of NS1, we confirm that the mechanisms reported here are novel and likely apply to early inflammatory responses occurring prior to NS1 expression. Importantly, while we showed that autophagy did not require NS1 to reduce IFN-β activity, inhibition of IFN-β by NS1 may rely in part on NS1-mediated autophagy stimulation in the infected cell^64^. Of note, even though we observed that autophagy competency did not impact the cell response to IFN-β within IAV-infected cells, autophagy perturbation may impact other cytokine pathways.

Future studies will be important to determine the role during the early phases of infection. As the inflammatory response to IAV impacts viral propagation and symptom severity, we suggest that autophagy-mediated suppression of inflammation represents a host mechanism to limit acute pathology.

## Materials and methods

### Cell lines, cell growth media, treatments and viruses

Atg5^+/+^ cells and Atg5^–/–^ MEFs were obtained from Christian Munz, University of Zurich, Switzerland. Atg7^–/–^ MEFs were obtained from Stephen Tait, University of Glasgow. All cell lines in this study were cultivated in complete growth medium, comprised of Dulbecco’s modified Eagle’s medium with high glucose, pyruvate and GlutaMAX and supplemented with non-essential amino-acids, HEPES buffer, penicillin/streptomycin (all reagents, ThermoFisher Scientific) and 10% foetal calf serum (GE Healthcare, A15-502). Shield1 (Clontech, 632188) was added at a final concentration of 1 µM (stock maintained at 1 mM) in growth media. Ethanol, the solvent for Shield1, was used as a control at a 1:1000 dilution in growth media. Treatments were used at the following concentrations: chloroquine (Sigma, C6628), 50 µM; PP242 (Selleck Chemical, S2218), 1 µM; MG132 (Sigma, C2211) 10 µM; recombinant IFN-β, at the indicated concentrations (BioLegend, 581302); and thapsigargin (Sigma, T9033) 3 µg/mL. Blocking IFNAR antibody (BD Pharmingen, 561183) or isotype control (BD Pharmingen, 553447) were used at 10 µg/mL and added to culture media 1 h before infection for the duration of the experiment. Influenza A/PR/8/76 (PR8) and ΔNS1 PR8 were purchased as purified allantoic fluid or purified antigen, respectively, from Charles River Laboratories (Spafas, CT, USA).

### Lentivirus production and clonal stably modified cell line generation

pLVX pTuner lentiviral vector (Clontech) with a puromycin resistance gene and destabilization domain at the 5’P end of the multiple cloning site was from Clontech. Mutagenesis PCR allowed the introduction of an AgeI site and 3 glycin codons (to increase flexibility between the DD and ATG5 protein) at the 5’-terminus of the ATG5 coding site in the pmCherry–ATG5 plasmid (Plasmid #13095; Addgene). Mutagenesis qPCR was performed using Phusion polymerase (ThermoFisher Scientific) following the manufacturer’s protocol and using primers: ACCGGTGGAGGAGGAACAGATGACAAAGATGTGCTT and CATGGTACCGTCGACTG. The ATG5^DD^ coding insert was then cleaved by AgeI and BamHI restriction enzymes and ligated into the pLVX pTuner lentiviral vector (all enzymes, New England Biolabs). The final plasmid (pATG5^DD^) was confirmed to have the expected sequence.

For the generation of the ATG5K130R^DD^ coding plasmid, mutagenesis PCR was performed using Phusion polymerase to change codon 130 from AAA to CGA in the pATG5^DD^ plasmid with the primers: CGAGAAGCTGATGCTTTAAAGCA and ATACACGACATAAAGTGAGCC. pBabeATG7^DD^, a retroviral vector coding for ATG7^DD^, was a generous gift from Douglas Green, St. Jude Children’s Research Hospital and Stephen Tait, University of Glasgow. Lentiviruses (from pATG5^DD^ and pATG5K130R^DD^) and retroviruses (pATG7^DD^) were produced and Atg5^–/–^ or Atg7^–/–^ MEFs were infected. Puromycin was used at 4 µg/mL for 1 week for selection (ThermoFisher Scientific, A11138-03) before single-cell cloning and phenotyping were performed.

### Virus infection

For infection, adherent cells were washed with growth medium without foetal calf serum. Growth medium, without serum, containing IAV or CHIKV was added to cells and plates were incubated at 37 °C for 1 h with gentle shaking every 15 min. Cells were then washed with complete growth medium before adding fresh complete growth medium with or without additional treatments.

### Immunoblotting

Cells were harvested by trypsinization, centrifuged and resuspended in 100 µL per million cells in lysis buffer: 1% Nonidet P40 substitute (Sigma, 74385) with protease inhibitor (Sigma, 11836145001). Protein concentration in the resulting supernatants was determined by BCA assay (ThermoFisher Scientific, 23225) following lysis on ice and clarification by centrifugation according to the manufacturer’s guidelines. In all, 30 µg of protein per sample were prepared in Lithium Dodecyl Sulphate sample buffer (ThermoFisher Scientific, NP0007) with dithiothreitol (20 mM final concentration) and loaded in 4–12% gradient polyacrylamide gel (Biorad, 3450124). Following transfer, polyvinylidene fluoride membranes (BioRad, 1704157) were blocked for 1 h in 5% w/v non-fat dry milk in TBS with 0.05% Tween 20 (Sigma P5927) (blocking solution). Membranes were then incubated for 15 h in 1:1000 dilution of the following antibodies in blocking solution with gentle shaking at 4 °C: anti-ATG5 (Abcam, ab108327), anti-GAPDH (CST, 2118), anti-LC3B (CST, 2775 S), anti-p62 (CST, 5114), anti-M2 (Abcam, ab5416), anti-IκBα (CST, 9242), and anti-PSMB10 (Abcam, ab77735) antibodies. Membranes were then incubated in 1:1000 horseradish peroxidase-conjugated anti-mouse antibody (CST, 7076 S) or anti-rabbit antibody (CST, 7074 S) in blocking solution for 1 h at room temperature with gentle shaking. Membranes were revealed with Supersignal Enhanced Chemoluminescence Substrate (ThermoFisher Scientific, 34080) according to the manufacturer’s guidelines and exposed to film.

### Intra-incubator microscopy

To monitor cell growth, cells were plated at 10,000 cells per well in 24-well plates. To monitor infection of cells by CHIKV harbouring a green fluorescent protein (GFP) coding sequence after the subgenomic promoter (a kind gift of Marco Vignuzzi, Institut Pasteur), 200,000 cells were plated in 12-well plates 24 h before infection. To monitor NF-κB transcription factor activity, cells were transfected with pNF-κB–GFP plasmid (a kind gift from Eliane Meurs, Institut Pasteur) using lipofectamine 2000 (ThermoFisher Scientific, 11668027) according to the manufacturer’s guidelines 24 h before plating (200,000 cells were plated in 12-well plates) and 40 h before infection. Images were taken every hour using Incucyte ZOOM System and analysis was performed using Incucyte ZOOM software (EssenBioScience).

### Flow cytometry and imaging flow cytometry

Cells were harvested and washed as for immunoblotting. After washing, cells were incubated for 20 min in 1:500 dilution of LIVE/DEAD Fixable Violet Dead Cell Stain (ThermoFisher, L34955) in phosphate-buffered saline (PBS), then 30 min in 1:100 antibody dilution for surface staining, all at 4 °C. Antibodies included allophycocyanin-conjugated anti-mouse CD274 (BD Pharmingen, 564715) or PE-conjugated anti-mouse H-2K^b^ (BD Pharmingen, 553570).

For intracellular staining (ICS), cells were fixed using BD Cytofix/Cytoperm Fixation and Permeabilization Solutions (BD Biosciences, 554722). Cells were then washed twice in BD Perm/wash buffer (PWB, BD Biosciences, 554723). Immunostaining was performed at 4 °C for 45 min with 1:100 dilution of fluorescein isothiocyanate (FITC)-conjugated anti-IAV NP antibody (Abcam, ab20921) or 1:500 dilution of anti-IAV M2 protein antibody (Abcam, ab5416) in PWB for 45 min at 4 °C. For M2 staining, cells were then incubated in 1:500 dilution of Alexa Fluor 647 goat anti-mouse IgG (H+L) (Jackson ImmunoResearch, 115-606-062) for 45 min at 4 °C. Cells were then washed in PWB buffer twice and in PBS once before resuspension in PBS and acquisition using a BD LSR Fortessa flow cytometer. Data analysis was performed using FlowJo 9 (Flowjo, LLC).

For imaging flow cytometry, the antibodies used for ICS were: mouse anti-LC3 antibody (MBL International) at 1:500 dilution and rabbit FITC-conjugated anti-IAV NP antibody (Abcam, ab20921) at 1:100 dilution. Staining of LC3 was performed before washing and staining with Alexa Fluor 647 goat anti-mouse IgG (H+L) (Jackson ImmunoResearch, 115-606-062). After washes, the anti-NP antibody was introduced. Cells were then washed in PWB buffer twice and in PBS once before resuspension in PBS and acquisition using Amnis Imagestream X imaging flow cytometer. Data analysis was performed using the Ideas software (Amnis); the masking strategy and the metric (bright detail intensity R3 or BDI R3) used to measure autophagic activity were previously described^65^.

### RNA extraction, RT and qPCR

For RNA extraction, cells were harvested and washed as for protein extraction. High pure RNA Isolation Kit (Roche, 11828665001) was used to extract RNA according to the provider’s protocol. For IAV RNA extraction from supernatants, 200 µL of supernatants cleared of cell debris by centrifugation at 1500 × *g* for 5 min at 4 °C were used instead of the 200 µL cell suspension in PBS at the first step of Roche’s protocol. RT was performed using Maxima reverse transcriptase (ThermoFisher Scientific, catalogue number: EP0741) and random primers (ThermoFisher Scientific, SO142). Taqman primer/probe mixes were used for cDNA quantification of *Hprt1* (Mm03024075_m1), *Ifnb1* (Mm00439546_s1), *Cd274* (Mm00452054_m1) and *Cxcl10* (Mm00445235_m1). For viral gene detection, we designed the following primer/probe sets:

**Table:** 

	Primer 1	Primer 2	FAM MGB probe
NS1	CACTGTGTCAAGCTTTCAGGTAGATT	GCGAAGCCGATCAAGGAAT	TTTCTTTGGCATGTCCG
M1	TCCAGTGCTGGTCTGAAAAATG	GGATCACTTGAACCGTTGCAT	AAAATTTGCAGGCCTATCA
M2	ACCGAGGTCGAAACGCCTAT	AAAAAAGACGATCAAGAATCCACAAT	TGCAGATGCAACGGT
NP	CGGAAAGTGGATGAGAGAACTCA	AGTCAGACCAGCCGTTGCAT	CCTTTATGACAAAGAAGAAA
PB1	TGTCAATCCGACCTTACTTTTCTTAA	TGTTGACAGTATCCATGGTGTATCC	CCAGCACAAAATG

Custom gene expression assays were synthesized by ThermoFisher Scientific. In all, 2 µL of cDNA diluted 4 times in water was used at concentration per qPCR reaction. qPCR was performed using Taqman Fast Advanced Master Mix (ThermoFisher Scientific) according to the provider’s protocol. StepOnePlus Real-Time PCR System (ThermoFisher Scientific) was used for thermocycling and data acquisition using the Fast Advanced Master Mix recommended conditions. The StepOnePlus software (ThermoFisher Scientific) was used for analysis.

### Haemagglutination assay

In all, 100 µL of two-fold serial dilutions (in PBS CaCl_2_^−^MgCl_2_^−^) of infected cell supernatants were mixed with 100 µL of 0.5% Guinea pig blood (Charles River Laboratories) in PBS CaCl_2_^−^MgCl_2_^−^ in round-bottom 96-well plates before incubation for 2 h at 4 °C. The haemagglutinin titre was calculated as the dilution of the most diluted well where red blood cell agglutination was visible.

### Power calculation for RT–qPCR

Standard deviation of Ct values did not show mean dependence. We therefore modelled Ct values of technical replicates for gene *i* as independent normally distributed random variables with mean *μ*_*i*_ and standard deviation *σ* (this latter parameter being independent of the gene). Estimation of *σ* was performed using pooled variance. RNA levels are compared using normalized data (ΔCt values), and by error propagation, ΔCt values follow a normal distribution with standard deviation *σ*’ = √2 *σ*. The power curve was computed using the function power.t.test in R (CRAN), with parameters sd = *σ*’, power = 0.8, sig.level = 0.05 and delta = log2(fold change).

### Nanostring nCounter assays, normalization and analysis

Infected cells were harvested and RNA was extracted as detailed above. nCounter Mouse Immunology Kit was used, following Nanostring guidelines. Raw RNA counts were exported from the nSolver Analysis Software (Nanostring). Raw RNA counts were normalized by housekeeping genes to account for the inter-sample variation of RNA quantity. The selected housekeeping gene pool was built from the 14 candidate control genes provided by Nanostring, following the geNorm method^39^. Briefly, for each two genes *j* ≠ *k*, pairwise variation coefficient *V*_*jk*_ is defined as

$$V_{{jk}}{\mathrm{ = }}\;{\mathrm{sd}}_i\left( {{\mathrm{log}}_2\left( {\frac{{a_{{ij}}}}{{a_{{ik}}}}} \right)} \right),$$

where *a*_*ij*_ is the number of counts for the gene *j* in the sample *i*. The gene stability measure *M*_*j*_ for control gene *j* is the arithmetic mean of all pairwise variations *V*_*jk*_ for *k* ≠ *j*. *M*_*j*_ evaluates the degree of correlation of gene *j* to other control genes (the smaller *M*_*j*_ is, the more correlated gene *j* is to other control genes). Genes were ranked by increasing *M* (Figure S2A), and to determine a threshold, the normalization factors NF_*n*_ was computed for all *n* (defined as the geometric mean of the housekeeping gene counts) of each sample when considering the *n* genes with lowest *M* as a housekeeping gene set (Figure S2B). Correlations between consecutive normalization factors increased then decreased when adding the sixth gene with lowest *M* (Figure S2C). This threshold was confirmed by studying the pairwise variation between consecutive NF_*n*_s (data not shown). The final housekeeping gene set consisted of the following five genes: *Ppia*, *Gapdh*, *Rpl19*, *Oaz1*, and *Polr2a*. Normalization was performed as follows: the scaling factor for a sample was defined as the ratio of the average across all geometric means and the geometric mean of the sample. For each sample, all gene counts were multiplied by the corresponding scaling factor.

As protein and mRNA data are generally close to log-normally distributed^66^, normalized RNA counts were subsequently log-transformed. For each time point, only genes that were consistently expressed above the lower limit of quantification were tested. Paired *t*-tests, which are shown to be extremely robust against non-normality^67^, were performed to compare Shield1-treated vs. untreated mRNA (log-transformed) normalized copy numbers. *z*-score was defined as

$$z = -{\mathrm{log}}_{10}\left( {p - {\mathrm{value}}} \right) \times \left| {{\mathrm{log}}_2\left( {{\mathrm{fold}}\;{\mathrm{change}}} \right)} \right|.$$

### Gene set enrichment analysis

Each gene was scored using the absolute value of the sum of the *t*-statistics from paired *t*-tests at 4 and 12 h. Genes were ranked by decreasing score, and gene set enrichment analysis was performed using GSEA v. 2.2.1 (Broad Institute), with the following settings: method, pre ranked gene list; gene setsdatabase, reactome (c2.cp.reactome.v5.1.symbols.gmt); number of permutations, 10,000; enrichment statistic, classic; min set size, 5; max set size, 100; and all other parameters as default.

### Quantitative approach to distinguish between IFN-β and IFN-γ signatures

Genes present in both nCounter panels (mouse immunology in this study and human immunology v.2 for the human whole blood study^41^) and consistently expressed above the lower limit of quantification were considered. Those for which the EtOH vs. Shield1 *t*-test *p*-value was <0.05 at 12 h were selected and weighted by their *t*-statistic, resulting in a 44-dimensional vector that was subsequently normalized by its L2 norm. Similar vectors were obtained by weighting the same 44 genes by the *t*-statistic from *t*-test comparing control vs. IFN-β or control vs. IFN-γ in the human whole blood study, after which they were also normalized. The difference 〈autophagy, IFN-β〉−〈autophagy, IFN-γ〉 between the scalar products was computed for 100,000 iterations through bootstrapping over the 25 donors of the whole-blood study.

### ELISA

CXCL10 ELISAs (R&D Biosystems, DY466) were performed using cell supernatant clarified by spinning at 850 × *g* for 5 min.

### Statistical analysis

Given that Shield1 treatment resulted in the reduced expression of ISGs (Fig. 3), statistical analysis in the subsequent figures (Figs. 4 and 5) was performed using one-tailed *t*-tests to test for downregulation of ISG expression upon Shield1 treatment. *t*-Tests were performed using GraphPad Prism 6 (Figs. 2, 4 and 5) or R (Fig. 3), and multiple testing correction was performed using R.

## Electronic supplementary material

Supplementary figures

Supplementary figure legends
