# Supplementary material for: Autophagy diminishes the early interferon-β response to influenza A virus resulting in differential expression of interferon-stimulated genes
Source: Cell Death Dis. 2018 May 10;9(5):539. doi: 10.1038/s41419-018-0546-5 (PMC5945842; doi:10.1038/s41419-018-0546-5)
Supplement: Supplementary file 1 — Supplementary figures [file 41419_2018_546_MOESM1_ESM.pdf]

Supplementary Figure 1

a

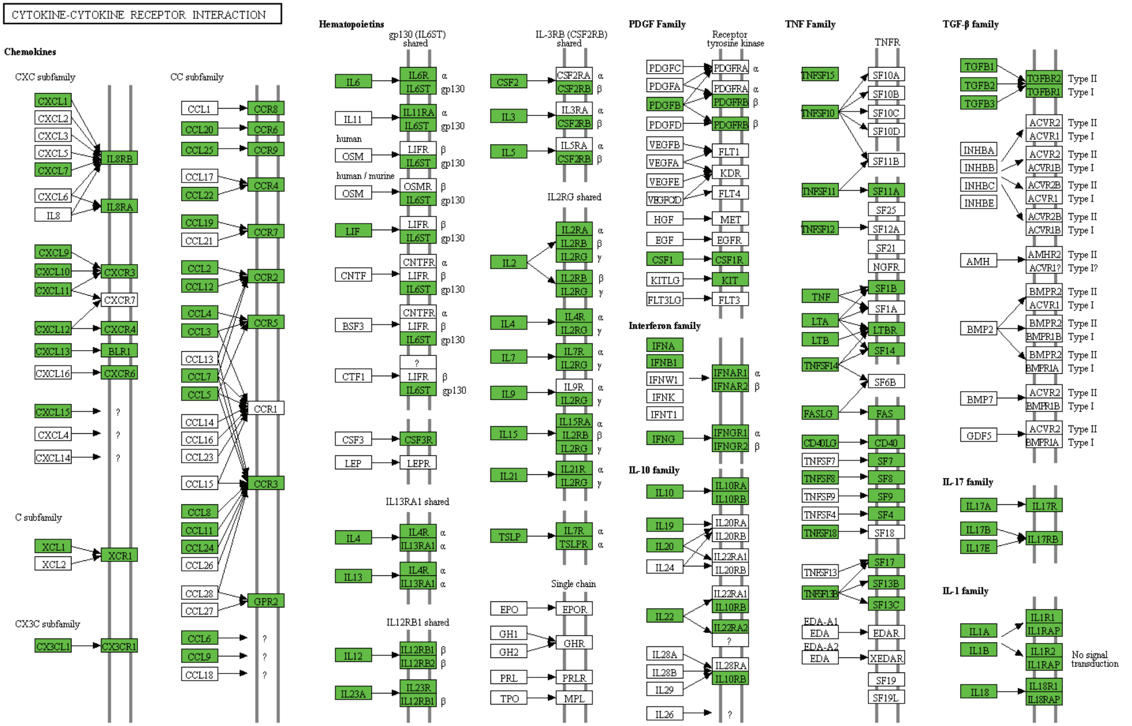

b

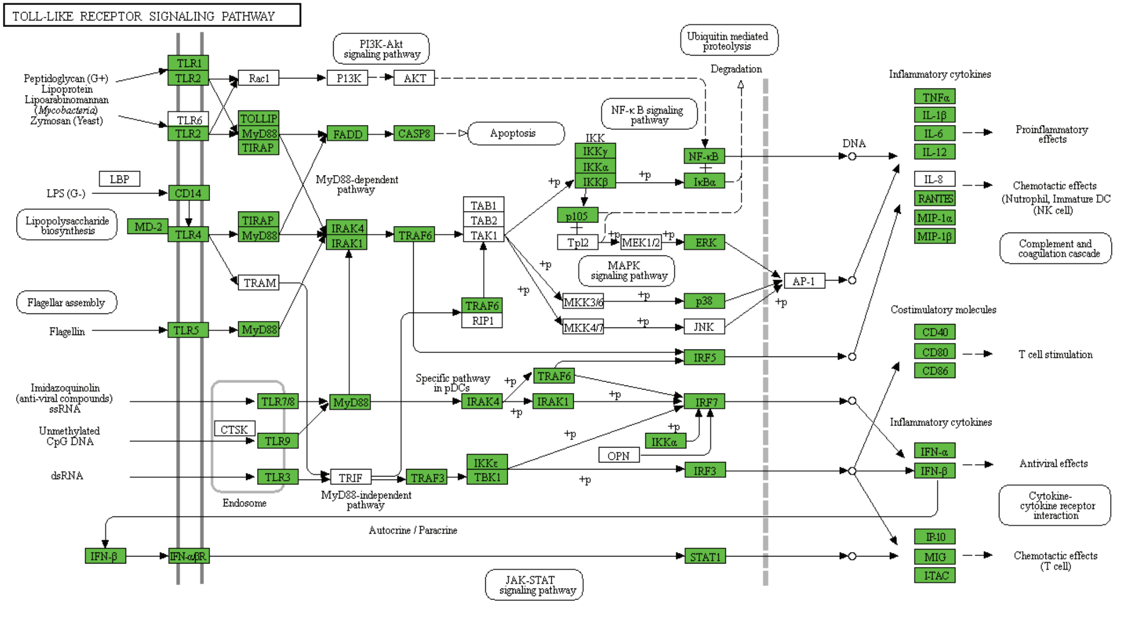

c

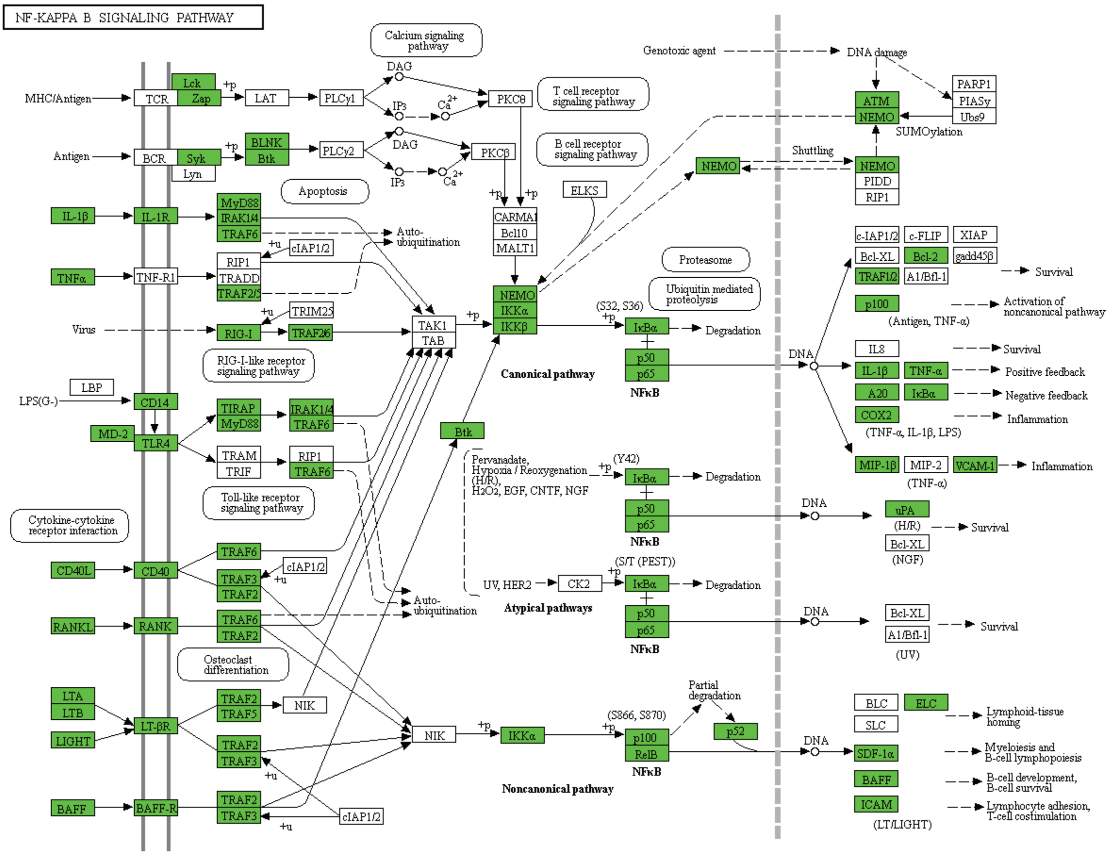
NF-KAPPA B SIGNALING PATHWAY
MHC/Antigen
TCR
Lck
Zap
LAT
PLCγ1
DAG
IP3
Ca2+
PKCθ
Calcium signaling pathway
T cell receptor signaling pathway
Antigen
BCR
Syk
Lyn
BLNK
Btk
PLCγ2
PKCβ
B cell receptor signaling pathway
Genotoxic agent
DNA damage
ATM
NEMO
PARP1
PIASy
Ubc9
SUMOylation
Shuttling
PIDD
RIP1
IL-1β
IL-1R
MyD88
IRAK1/4
TRAF6
Apoptosis
Auto-ubiquitination
ELKS
CARMA1
Bcl10
MALT1
TNFα
TNF-R1
TRADD
TRAF2/5
cIAP1/2
IKKα
IKKβ
Proteasome
Ubiquitin mediated proteolysis
Virus
RIG-I
TRIM25
TAK1
TAB
(S32, S36)
IκBα
Degradation
p50
p65
NFκB
Canonical pathway
RIG-I-like receptor signaling pathway
LBP
LPS(G-)
CD14
MD-2
TLR4
TIRAP
TRAM
TRIF
Btk
Toll-like receptor signaling pathway
Cytokine-cytokine receptor interaction
Pervanadate, Hypoxia / Reoxygenation (H/R), H2O2, EGF, CNTF, NGF
(Y42)
CD40L
CD40
TRAF3
TRAF2
UV, HER2
CK2
(S/T (PEST))
Atypical pathways
RANKL
RANK
Osteoclast differentiation
LTA
LTB
LIGHT
LT-βR
NIK
IKKα
(S866, S870)
Partial degradation
p100
RelB
p52
Noncanonical pathway
BAFF
BAFF-R
DNA
c-IAP1/2
c-FLIP
XIAP
Bcl-XL
Bcl-2
gadd45β
TRAF1/2
A1/Bfl-1
Survival
Activation of noncanonical pathway
(Antigen, TNF-α)
IL8
IL-1β
TNF-α
A20
IκBα
COX2
Positive feedback
Negative feedback
Inflammation
(TNF-α, IL-1β, LPS)
MIP-1β
MIP-2
VCAM-1
(TNF-α)
uPA
(H/R)
(NGF)
(UV)
BLC
ELC
SLC
SDF-1α
ICAM
(LT/LIGHT)
Lymphoid-tissue homing
Myeloiesis and B-cell lymphopoiesis
B-cell development, B-cell survival
Lymphocyte adhesion, T-cell costimulation

d

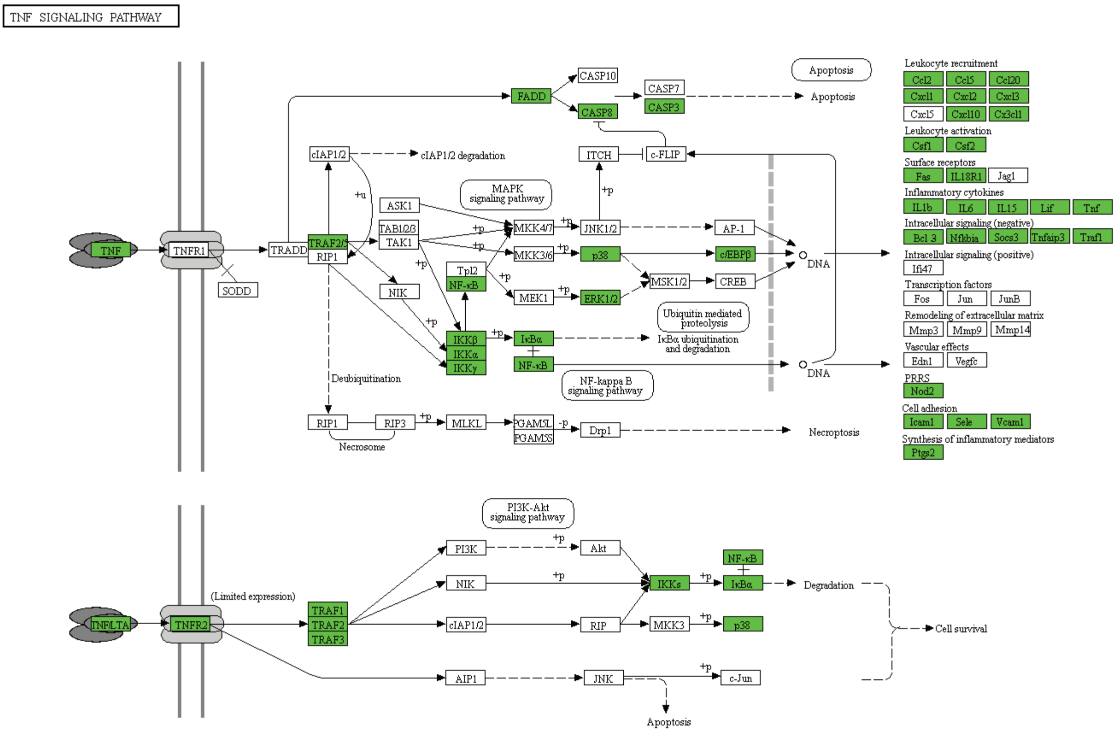
TNF SIGNALING PATHWAY
TNF
TNFR1
SODD
TRADD
TRAF2/5
RIP1
cIAP1/2
cIAP1/2 degradation
FADD
CASP10
CASP8
CASP7
CASP3
Apoptosis
ITCH
c-FLIP
MAPK signaling pathway
ASK1
TAB1/2/3
TAK1
MKK4/7
MKK3/6
JNK1/2
p38
AP-1
c/EBPβ
Tpl2
NF-κB
MEK1
ERK1/2
MSK1/2
CREB
DNA
NIK
IKKβ
IKKα
IKKγ
IκBα
Ubiquitin mediated proteolysis
IκBα ubiquitination and degradation
NF-kappa B signaling pathway
Deubiquitination
RIP3
MLKL
PGAM5L
PGAM5S
Drp1
Necroptosis
Necrosome
Leukocyte recruitment
Ccl2
Ccl5
Ccl20
Cxcl1
Cxcl2
Cxcl3
Cxcl5
Cxcl10
Cx3cl1
Leukocyte activation
Csf1
Csf2
Surface receptors
Fas
IL18R1
Jag1
Inflammatory cytokines
IL1b
IL6
IL15
Lif
Tnf
Intracellular signaling (negative)
Bcl 3
Nfkbia
Socs3
Tnfaip3
Traf1
Intracellular signaling (positive)
Ifi47
Transcription factors
Fos
Jun
JunB
Remodeling of extracellular matrix
Mmp3
Mmp9
Mmp14
Vascular effects
Edn1
Vegfc
PRRS
Nod2
Cell adhesion
Icam1
Sele
Vcam1
Synthesis of inflammatory mediators
Ptgs2
PI3K-Akt signaling pathway
PI3K
Akt
IKKs
Degradation
(Limited expression)
TNF/LTA
TNFR2
TRAF1
TRAF2
TRAF3
RIP
MKK3
Cell survival
AIP1
JNK
c-Jun

Supplementary Figure 2

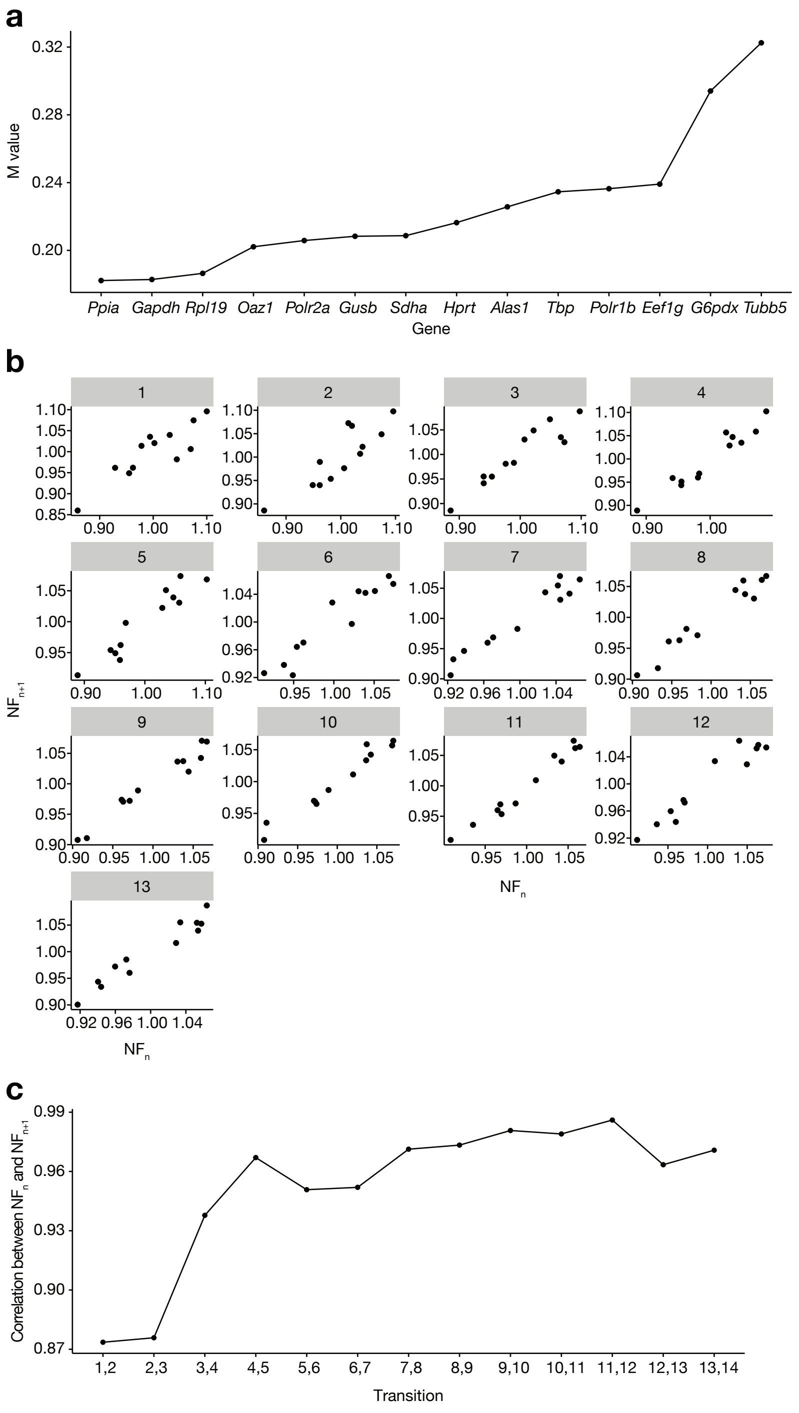

Supplementary Figure 3

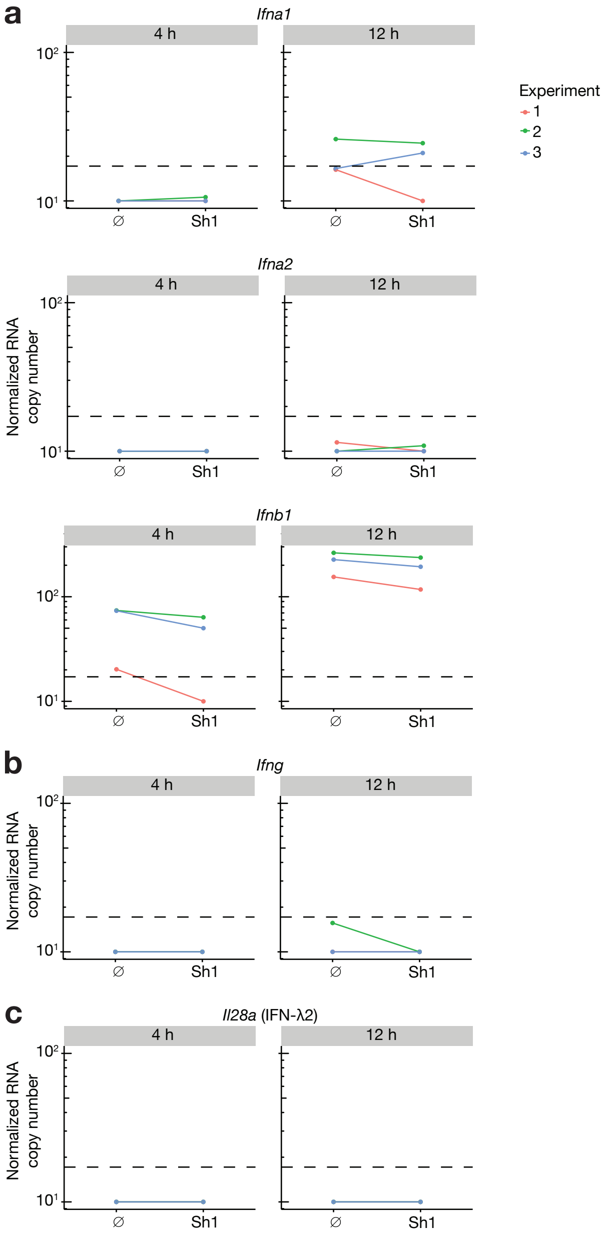

Supplementary Figure 4

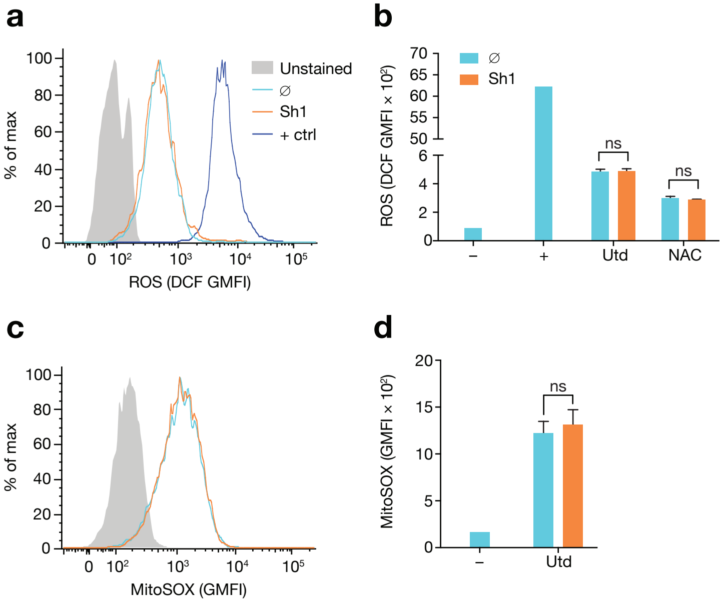

Supplementary Figure 5

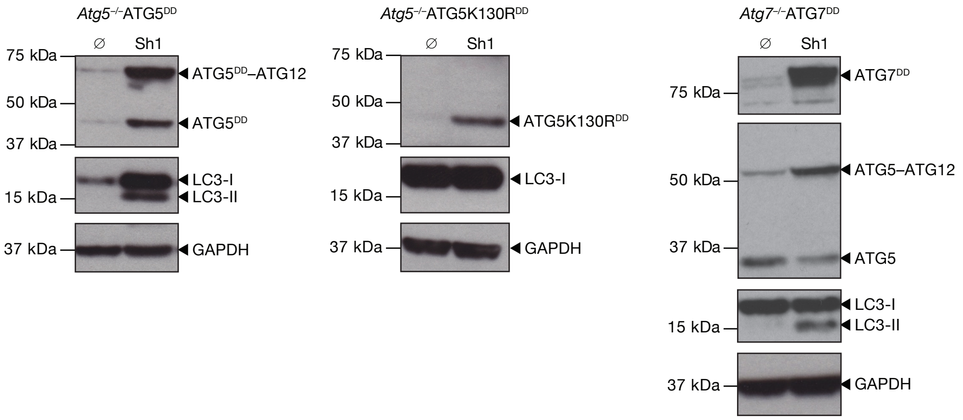

Supplementary Figure 6

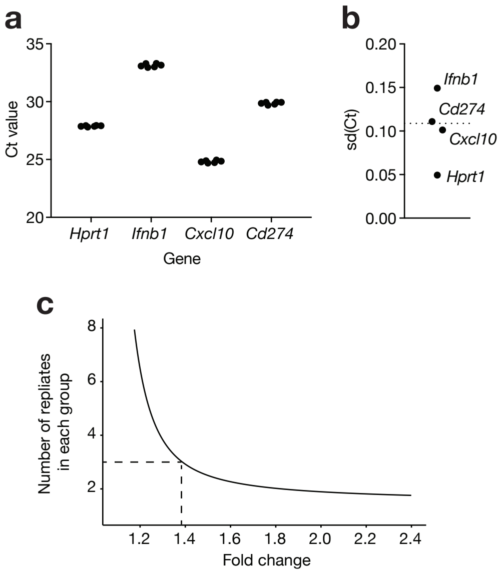

Table S1

4 h

	Gene	t-statistic	p-value	q-value	FC	z-value
1	*Cd3eap*	-2730.5	1.34E-07	3.25E-05	0.88276	1.236401
2	*Cd24a*	-77.591	0.00017	0.02009	0.87429	0.732589
3	*Il6st*	27.9668	0.00128	0.10294	1.03372	0.138452
4	*Cxcl10*	13.6059	0.00536	0.30754	1.40089	1.10446
5	*Ahr*	-12.371	0.00647	0.30754	0.88044	0.402119
6	*Tlr3*	10.2275	0.00943	0.30754	1.31015	0.789482
7	*Maf*	-9.0192	0.01207	0.30754	0.87179	0.379728
8	*Il1rn*	-8.5344	0.01345	0.30754	0.85855	0.411692
9	*Psmd7*	-8.0044	0.01525	0.30754	0.98005	0.052827
10	*Nfkbiz*	7.86164	0.0158	0.30754	1.08673	0.21615
11	*Notch2*	-7.5673	0.01702	0.30754	0.96737	0.084681
12	*Pml*	7.28403	0.01833	0.30754	1.1731	0.400024
13	*Cd274*	6.82393	0.02081	0.30754	1.63318	1.190183
14	*Ifih1*	6.58922	0.02227	0.30754	1.21285	0.460012
15	*Il6*	6.47391	0.02304	0.30754	1.3499	0.708813
16	*Mx1*	6.04646	0.02628	0.30754	1.37267	0.72221
17	*Tgfbi*	-5.9061	0.02749	0.30754	0.72959	0.709921
18	*Itga6*	-5.9053	0.0275	0.30754	0.9387	0.142445
19	*Klra4*	5.77515	0.0287	0.30754	1.13367	0.279135
20	*Tlr2*	5.57668	0.03068	0.30754	1.16966	0.342093
21	*Casp2*	5.37019	0.03297	0.30754	1.07981	0.164166
22	*Cd80*	5.3628	0.03306	0.30754	1.03687	0.077348
23	*Tapbp*	5.35903	0.0331	0.30754	1.17895	0.351538
24	*B2m*	5.18974	0.03518	0.30754	1.06641	0.13485
25	*Socs1*	5.07328	0.03673	0.30754	1.18343	0.348672
26	*C1qbp*	-5.0347	0.03726	0.30754	0.96165	0.080605
27	*Ccl2*	5.0334	0.03728	0.30754	1.15082	0.28951
28	*Atg16l1*	-5.0156	0.03753	0.30754	0.96668	0.069706
29	*Irgm1*	4.96889	0.0382	0.30754	1.23329	0.428957
30	*Il15ra*	4.94069	0.03861	0.30754	1.22106	0.407223
31	*Ifit2*	4.83984	0.04014	0.30754	1.24129	0.435467
32	*Gpi1*	-4.8063	0.04067	0.30754	0.83989	0.350099
33	*Gm10499*	4.3207	0.04961	0.3274	1.29568	0.48747
34	*Cd109*	-4.3139	0.04976	0.3274	0.98599	0.026525
35	*Ddx58*	4.26495	0.05082	0.3274	1.1133	0.200364
36	*Tap1*	4.26323	0.05086	0.3274	1.27006	0.446167
37	*Ifi204*	4.20985	0.05206	0.3274	1.1944	0.328953
38	*Lef1*	-4.1638	0.05313	0.3274	0.81642	0.373004
39	*Tgfb1*	-4.0411	0.05613	0.3274	0.94348	0.104992
40	*Igf2r*	-4.0245	0.05656	0.3274	0.84591	0.301184
41	*Ikbkg*	4.01273	0.05686	0.3274	1.19221	0.315832
42	*Stat1*	3.9639	0.05815	0.3274	1.23012	0.369159
43	*Bst2*	3.96293	0.05817	0.3274	1.14567	0.242351
44	*Irf7*	3.88165	0.06042	0.33229	1.20254	0.324318
45	*Bcl2*	-3.712	0.06552	0.34629	0.86214	0.253307
46	*Itgb1*	-3.6637	0.06709	0.34629	0.98518	0.025282
47	*Mif*	-3.6432	0.06777	0.34629	0.97767	0.038087
48	*Stat3*	3.61622	0.06868	0.34629	1.05345	0.087374
49	*Tollip*	-3.4918	0.07313	0.35066	0.90736	0.159303
50	*Stat2*	3.47182	0.07389	0.35066	1.39396	0.542174
51	*Il1r1*	-3.3575	0.07842	0.35066	0.9185	0.135593
52	*Pdgfrb*	-3.3435	0.079	0.35066	0.7443	0.469668
53	*Myd88*	3.27874	0.08177	0.35066	1.13395	0.197204
54	*Irf1*	3.26307	0.08247	0.35066	1.17154	0.247532
55	*Crlf2*	-3.2458	0.08324	0.35066	0.89882	0.166162
56	*Ifi35*	3.24024	0.08349	0.35066	1.1267	0.185595
57	*Fas*	3.22168	0.08434	0.35066	1.16529	0.237022
58	*Cxcl12*	-3.2083	0.08496	0.35066	0.93781	0.099184
59	*Tfrc*	3.19679	0.08549	0.35066	1.0405	0.061182
60	*Casp3*	3.14787	0.08783	0.35423	1.07413	0.108985
61	*Jak2*	3.10007	0.0902	0.35783	1.0895	0.129207
62	*Hif1a*	3.00506	0.09519	0.37154	1.02316	0.033739
63	*H2.DMa*	2.91815	0.10011	0.3716	1.21577	0.281739
64	*Phlpp2*	-2.8892	0.10183	0.3716	0.95988	0.058602
65	*Pdgfb*	-2.8823	0.10224	0.3716	0.77622	0.361935
66	*Tnfaip6*	2.88179	0.10227	0.3716	1.18644	0.244228
67	*Fn1*	-2.8718	0.10288	0.3716	0.93414	0.097083
68	*Traf5*	2.8318	0.10536	0.37496	1.15553	0.203821
69	*C1ra*	-2.7875	0.10821	0.37951	0.90497	0.139122
70	*Tnfsf10*	2.72291	0.11256	0.38701	1.22429	0.276943
71	*Il10rb*	-2.7087	0.11354	0.38701	0.94591	0.075798
72	*Jak3*	2.6717	0.11618	0.39028	1.16211	0.20263
73	*Ptger4*	-2.623	0.11978	0.39028	0.82402	0.257357
74	*Il6ra*	-2.6168	0.12025	0.39028	0.96927	0.041424
75	*Pdcd2*	-2.6076	0.12095	0.39028	0.89553	0.146033
76	*Pparg*	-2.527	0.12736	0.40435	0.91202	0.118905
77	*Prkcd*	-2.5114	0.12866	0.40435	0.95956	0.053036
78	*Ly96*	2.44426	0.13444	0.40688	1.09302	0.111833
79	*Irf5*	2.44407	0.13445	0.40688	1.16408	0.191006
80	*Ifngr1*	-2.4309	0.13563	0.40688	0.98758	0.015649
81	*Bcl6*	-2.4248	0.13619	0.40688	0.9085	0.119879
82	*Casp8*	-2.3741	0.14087	0.41575	0.95244	0.05984
83	*Cd99*	-2.318	0.14634	0.42667	0.93022	0.087103
84	*Tnfrsf1b*	2.27972	0.15023	0.4328	1.08917	0.101443
85	*Npc1*	-2.2544	0.15288	0.43416	0.96818	0.038053
86	*Ikbkap*	-2.2412	0.15429	0.43416	0.98277	0.020356
87	*Psmb10*	2.21713	0.15691	0.43646	1.21087	0.22204
88	*Mme*	-2.158	0.1636	0.4499	0.83527	0.204167
89	*Tyk2*	-2.1363	0.16616	0.4518	0.84547	0.188768
90	*Runx1*	2.11159	0.16913	0.45477	1.0637	0.068756
91	*Rae1*	-2.0481	0.17712	0.46199	0.97849	0.023583
92	*H2.K1*	2.04567	0.17743	0.46199	1.15052	0.151912
93	*Il1rap*	2.03858	0.17835	0.46199	1.06075	0.063702
94	*Fyn*	-2.0302	0.17945	0.46199	0.96255	0.041081
95	*Bax*	-2.0037	0.18299	0.46505	0.9573	0.046431
96	*Ptk2*	1.99284	0.18448	0.46505	1.04502	0.046636
97	*Litaf*	1.96998	0.18765	0.46816	1.04183	0.042957
98	*Plaur*	-1.9321	0.19307	0.47676	0.96295	0.038907
99	*Jak1*	-1.8753	0.20158	0.48504	0.98846	0.011647
100	*Ikbke*	1.84257	0.20672	0.48504	1.10617	0.099663

12 h

	Gene	t-statistic	p-value	q-value	FC	z-value
1	*Cd274*	27.0385	0.00137	0.09074	1.54174	1.789278
2	*Ctsc*	24.6834	0.00164	0.09074	1.08992	0.346062
3	*Irf1*	24.4996	0.00166	0.09074	1.23335	0.840984
4	*Psmb10*	24.4885	0.00166	0.09074	1.35075	1.205411
5	*Ly96*	-20.391	0.0024	0.09074	0.80019	0.842688
6	*Ifngr2*	19.2717	0.00268	0.09074	1.07263	0.260134
7	*Csf1*	19.1991	0.0027	0.09074	1.10386	0.366137
8	*Ahr*	-18.443	0.00293	0.09074	0.94623	0.202037
9	*Pml*	14.8064	0.00453	0.12484	1.12305	0.392405
10	*Muc1*	13.3608	0.00556	0.13777	1.14852	0.450565
11	*Ccl5*	12.0997	0.00676	0.14661	1.31962	0.868259
12	*Irf3*	11.8096	0.00709	0.14661	1.32068	0.862403
13	*Ptpn22*	9.75634	0.01034	0.19038	1.4811	1.12504
14	*Ifi35*	9.56828	0.01075	0.19038	1.07836	0.214275
15	*Tapbp*	8.22536	0.01446	0.23154	1.19179	0.465719
16	*Tnfsf10*	7.83174	0.01592	0.23154	1.2706	0.621295
17	*Il1rap*	7.61022	0.01683	0.23154	1.09116	0.223263
18	*Vcam1*	7.54852	0.0171	0.23154	1.43893	0.927649
19	*Nfkbia*	7.06461	0.01945	0.23154	1.09894	0.23288
20	*Nfil3*	6.89357	0.0204	0.23154	1.12627	0.289972
21	*Irak1*	-6.8707	0.02053	0.23154	0.94913	0.127102
22	*Abcb10*	6.65965	0.02181	0.23154	1.06262	0.145579
23	*Zbtb7b*	-6.3467	0.02394	0.23154	0.87822	0.303674
24	*Ifngr1*	6.12621	0.02563	0.23154	1.0371	0.083621
25	*Ptpn6*	6.01936	0.02651	0.23154	1.34012	0.66591
26	*Tap1*	5.7224	0.02921	0.23154	1.28738	0.559235
27	*Eef1g*	-5.7087	0.02934	0.23154	0.975	0.055981
28	*H2.K1*	5.50763	0.03142	0.23154	1.17989	0.358643
29	*Tfrc*	5.45089	0.03205	0.23154	1.18185	0.360174
30	*Ifi204*	5.31563	0.03362	0.23154	1.2553	0.483335
31	*Fyn*	-5.2626	0.03426	0.23154	0.91158	0.195688
32	*Cd74*	5.25979	0.0343	0.23154	1.22115	0.422189
33	*B2m*	5.24762	0.03445	0.23154	1.09006	0.181987
34	*Ifih1*	5.13278	0.03592	0.23154	1.15392	0.29838
35	*Stat1*	5.12504	0.03603	0.23154	1.13328	0.260534
36	*Tnfaip6*	5.00762	0.03764	0.23154	1.37595	0.655798
37	*Irgm1*	4.91887	0.03893	0.23154	1.18558	0.34621
38	*Tlr2*	4.89733	0.03926	0.23154	1.35169	0.611321
39	*Tollip*	-4.816	0.04051	0.23154	0.86409	0.293447
40	*Tlr3*	4.76894	0.04127	0.23154	1.42878	0.712667
41	*Il13ra1*	4.72633	0.04197	0.23154	1.21507	0.387015
42	*Lif*	4.66255	0.04305	0.23154	1.30324	0.521964
43	*Cd44*	4.62044	0.04379	0.23154	1.0484	0.092653
44	*Irf7*	4.61641	0.04386	0.23154	1.15654	0.284911
45	*Atm*	4.61556	0.04387	0.23154	1.09359	0.175258
46	*Cxcl10*	4.56344	0.04482	0.23154	1.46645	0.744853
47	*Bcl6*	4.52262	0.04557	0.23154	1.11209	0.205576
48	*Ddx58*	4.51636	0.04569	0.23154	1.1003	0.184798
49	*Cebpb*	-4.5134	0.04575	0.23154	0.9007	0.202123
50	*Bax*	-4.4083	0.0478	0.23709	0.94097	0.115916
51	*Lck*	4.31939	0.04964	0.24139	1.23334	0.394592
52	*Pdcd2*	-4.2327	0.05154	0.24158	0.93437	0.126116
53	*Pou2f2*	4.19193	0.05247	0.24158	1.2543	0.418438
54	*Il6st*	4.18616	0.0526	0.24158	1.07495	0.133352
55	*Nfkb2*	4.0054	0.05705	0.25724	1.04612	0.080902
56	*Tmem173*	3.91985	0.05935	0.26072	1.16342	0.267848
57	*Fcgr2b*	3.86375	0.06093	0.26072	1.2702	0.419301
58	*Il6*	3.86215	0.06097	0.26072	1.54922	0.767232
59	*Ccl2*	3.77162	0.06366	0.26758	1.33275	0.495681
60	*Cxcl11*	3.73072	0.06493	0.26837	1.7343	0.943343
61	*Ski*	-3.6561	0.06734	0.2703	0.92041	0.140201
62	*Ncam1*	-3.6491	0.06757	0.2703	0.88338	0.20935
63	*Tgfb1*	-3.5938	0.06946	0.27186	0.95151	0.083065
64	*Ifit2*	3.5738	0.07016	0.27186	1.18303	0.279807
65	*Ifnb1*	3.5292	0.07175	0.27377	1.1967	0.296408
66	*Npc1*	-3.4362	0.07526	0.2799	0.77645	0.410104
67	*Cxcl1*	3.42692	0.07562	0.2799	1.56208	0.72156
68	*Igf2r*	-3.3691	0.07794	0.28107	0.89557	0.176346
69	*Stat2*	3.36274	0.0782	0.28107	1.24358	0.348081
70	*Itgb1*	-3.2336	0.08379	0.28901	0.96121	0.061467
71	*Fadd*	-3.2046	0.08512	0.28901	0.91115	0.143637
72	*Tnfaip3*	3.20323	0.08519	0.28901	1.20207	0.283998
73	*Ptgs2*	3.19396	0.08562	0.28901	1.162	0.231208
74	*Nfatc3*	3.16889	0.08681	0.28901	1.04441	0.066545
75	*Plaur*	3.15663	0.0874	0.28901	1.05977	0.088656
76	*Stat3*	3.08119	0.09116	0.29747	1.0723	0.104764
77	*H2.DMa*	3.02586	0.09406	0.30248	1.11307	0.158652
78	*Fn1*	-3.0004	0.09544	0.30248	0.91486	0.130978
79	*Mx1*	2.98023	0.09656	0.30248	1.44771	0.541888
80	*Ets1*	2.96215	0.09757	0.30248	1.12407	0.170533
81	*Fcgrt*	2.93505	0.09912	0.30349	1.04973	0.070288
82	*Cd99*	-2.9045	0.10091	0.30519	0.95056	0.072856
83	*Runx1*	2.8701	0.10298	0.30771	1.06598	0.091003
84	*App*	-2.8275	0.10563	0.31186	0.93325	0.097297
85	*Nfkb1*	2.77921	0.10875	0.3173	1.07285	0.097756
86	*Hlx*	2.75226	0.11055	0.31841	1.04624	0.06237
87	*Itga2b*	2.73536	0.1117	0.31841	1.18339	0.231258
88	*Tyk2*	2.68412	0.11529	0.32057	1.13083	0.166417
89	*Tslp*	2.67085	0.11624	0.32057	1.41754	0.470484
90	*Tbk1*	-2.6696	0.11634	0.32057	0.91726	0.116416
91	*Ptpn2*	2.63006	0.11925	0.32217	1.03161	0.041462
92	*Mapk11*	2.60032	0.12152	0.32217	1.19203	0.231976
93	*C1qbp*	-2.5856	0.12266	0.32217	0.99192	0.010663
94	*Stat5a*	-2.5507	0.12543	0.32217	0.84227	0.223276
95	*Psmb9*	2.51895	0.12803	0.32217	1.36015	0.396152
96	*Stat6*	-2.5184	0.12807	0.32217	0.92466	0.100856
97	*Tlr4*	-2.5083	0.12891	0.32217	0.90911	0.12231
98	*Gpi1*	2.50465	0.12922	0.32217	1.13822	0.16598
99	*Ccl7*	2.49864	0.12973	0.32217	1.19287	0.225676
100	*Prim1*	-2.4896	0.13049	0.32217	0.9383	0.081264
