# Supplementary material for: Autophagy diminishes the early interferon-β response to influenza A virus resulting in differential expression of interferon-stimulated genes
Source: Cell Death Dis. 2018 May 10;9(5):539. doi: 10.1038/s41419-018-0546-5 (PMC5945842; doi:10.1038/s41419-018-0546-5)
Supplement: Supplementary file 2 — Supplementary figure legends [file 41419_2018_546_MOESM2_ESM.docx]

**Supplementary figure legends**

**Figure S1: Gene expression pathways represented in the NanoString Mouse Immunology panel.** KEGG database pathway analysis of Cytokine-Cytokine Receptor (**a**), NF-κB (**b**), TNFA (**c**) and TLR (**d**) signalling pathways, with genes included in the NanoString analysis coloured in green. Genes in white were not represented on the NanoString code set.

**Figure S2: Strategy for selection of internal control genes following the geNorm method**. (**a**) The 14 candidate control genes provided by Nanostring were ordered by increasing M value, i.e., by decreasing correlation with the other candidate control genes (see Materials and Methods). (**b** to **c**) Normalization factors arising from addition of one control gene were compared for each addition (**b**), and consecutive Pearson’s correlation coefficients were computed and are shown in the graph (**c**).

**Figure S3: Only *Ifnb1* RNA is consistently expressed by ATG5^DD^ cells after IAV PR8 infection.** ATG5^DD^ cells, pre-treated for 16 h with Shield1 (Sh1), were infected with IAV PR8 at MOI 3 for 4 or 12 h after which RNA was extracted. RNA levels were quantified using Nanostring nCounter technology. Expression of type I (**a**), type II (**b**), and type III (**c**) interferons are depicted. Dashed lines represent the lower limit of quantification of the assay. A threshold of 10 was used for visualization purposes, hence points below 10 overlap at 10.

**Figure S4: ATG5^DD^ stabilization does not impact cellular ROS levels.** **(a, b)** ATG5^DD^-expressing ATG5^–/–^ cells, treated or not with Shield1 (Sh1) for 24 h were treated with 20 µM DCFDA in the last 4 h of Sh1 treatment with or without N-acetyl-cysteine (NAC), an antioxidant. DCF fluorescence was monitored by flow cytometry representative histograms **(a)** and bar graph **(b)** are shown. **(c, d)** Mitochondrial ROS were monitored by flow cytometry using mitoSOX after treating ATG5^DD^-expressing ATG5^–/–^ cells with Sh1 or vehicle for 24 h. Representative histograms **(c)** and bar graph **(d)** are shown. Graphs show mean and standard deviation of triplicates, and data are representative of two experiments. ns, not significant (one-tailed unpaired *t*-test followed by Holm’s multiple testing correction).

**Figure S5: Stabilization of ATG7^DD^ in *Atg7*^–/–^ cells but not of ATG5K130R^DD^ in *Atg5*^–/–^ cells induces autophagy capacity.** *Atg5*^–/–^ cells expressing ATG5^DD^, *Atg5*^–/–^ cells expressing ATG5K130R^DD^, or *Atg7*^–/–^ cells expressing ATG7^DD^, were treated or not with Shield1 (Sh1) for 20 h before immunoblotting was performed using anti-ATG5, anti-ATG7, anti-LC3, and anti-GAPDH antibodies.

**Figure S6: Detection power of RT–qPCR for *Hprt1*, *Ifnb1*, *Cxcl10* and *Cd274*.
(a** and **b)** ATG5^DD^ cells treated with Shield1 were infected with IAV at MOI 3 for 4 h before RNA extraction. For each RNA product, 6 different RT–qPCRs were performed for the specified gene. Threshold cycle (Ct) values (**a**) and standard deviation of Ct are shown for each gene (**b**). (**c**) After estimation of the standard deviation of ΔCt values, shown is the power curve giving the number of technical replicates required to detect different fold changes with 80% power at a confidence level of 95%; e.g., 3 replicates allow the detection of a ~1.4-fold change (dashed line).

**Table S1: Genes most impacted by autophagy capacity.** *p*-values were determined by two-tailed paired *t*-tests comparing control vs Shield1-treated cells, and *z*-values were subsequently computed (see Materials and Methods for formula). *q*-values correspond to false-discovery rate. The 100 genes with smallest p-values at 4 h and 12 h post-infection are listed. In red are the 44 genes, present in the gene sets of both this study and the whole-blood study,^41^ that were weighted by their *t*-statistic to distinguish between a type I and a type II interferon response, with *p*-value < 0.05 at 12 h post-infection. FC, fold change.
